# High-definition MEG source estimation using the reciprocal boundary element fast multipole method

**DOI:** 10.1016/j.neuroimage.2025.121452

**Published:** 2025-09-20

**Authors:** Guillermo Núñez Ponasso, Derek A. Drumm, Abbie Wang, Gregory M. Noetscher, Matti Hämäläinen, Thomas R. Knösche, Burkhard Maess, Jens Haueisen, Sergey N. Makaroff, Tommi Raij

**Affiliations:** a Department of Electrical & Computer Engineering, Worcester Polytechnic Institute, Worcester, MA, USA; b Graduate School of Information Sciences, Division of Mathematics, Tohoku University, Sendai, Miyagi, Japan; c Department of Neuroscience and Biomedical Engineering, Aalto University, Espoo, Finland; d Max Planck Institute for Cognitive and Brain Sciences, Leipzig, Germany; e Technische Universität Ilmenau, Ilmenau, Germany; f Athinoula A. Martinos Center for Biomedical Imaging, Department of Radiology, Massachusetts General Hospital, Boston, MA, USA

## Abstract

*Magnetoencephalographic* (MEG) source estimation relies on the computation of the gain (lead-field) matrix, which embodies the linear relationship between the amplitudes of the sources and the recorded signals. However, with a realistic forward model, the calculation of the gain matrix in a “direct” fashion is a computationally expensive task, forcing the number of dipolar sources in standard MEG pipelines to be typically limited to ~10,000. We propose a fast computational approach to calculate the gain matrix, which is based on the reciprocal relationship between MEG and transcranial magnetic stimulation (TMS), and which we couple with the charge-based boundary element fast multipole method (BEM-FMM). Our method allows us to efficiently generate gain matrices for high-resolution multi-layer non-nested meshes involving source spaces of up to ~1 million dipoles. We employed the gain matrices generated with our approach to perform minimum norm estimate (MNE) source localization against simulated data (at varying noise levels) and experimental MEG data of evoked somatosensory fields elicited by right-hand median nerve stimulation on 5 healthy participants. Additionally, we compare our experimental source estimates against the standard 1- and 3-layer BEM models of the MNE-Python source estimation pipeline, and against a 3-layer isotropic FEM model.

## 1. Introduction

Source estimation (Knösche and Haueisen, 2022) (also known as source localization, or source reconstruction) aims to estimate neural activity sites and time courses from non-invasive measurements such as *electroencephalography* (EEG) and *magnetoencephalography* (MEG) (Hämäläinen et al., 1993). This technique can be used clinically to diagnose pathologies, for example, by localizing sources of interictal epileptic activity (Plummer et al., 2008), or studying ADHD (Jonkman et al., 2004); it also has basic-research applications in mapping higher cognitive function (Siebenhühner et al., 2016; Gross, 2019), in psychological and psychiatric research (Foti et al., 2011; Hämäläinen et al., 2015; Uhlhaas et al., 2017), and as a component in brain-computer interfaces (Noirhomme et al., 2008).

The methodology of MEG/EEG source estimation can be split into two main components: the forward model and the inverse model. The forward model depends on subject anatomy, sensor placement and specifications, and assumptions on conductivity values. In this step, sensor outputs are calculated for each individual putative source, each modeled as a current dipole (Hämäläinen et al., 1993) , by solving the quasistatic Maxwell equations (Nuñez Ponasso, 2024). An important point is that the only sources of indeterminacy in the forward stage are due to MRI segmentation errors (Lanfer et al., 2012), uncertainty of conductivity values (Homma et al., 1995; Haueisen et al., 1999; Vorwerk et al., 2024), and co-registration errors (Chella et al., 2019); any other errors are numerical.

On the other hand, the inverse problem is ill posed. Even though an infinite number of possible solutions exist (Helmholtz, 1853), useful and accurate estimates of the source locations can be obtained by introducing biophysical constraints. Examples of such assumptions are: (1) limiting the source model to the interface between gray and white matter; (2) assuming that equivalent current dipoles are oriented perpendicular to the gray matter surface (Nunez, 1981); or (3) assumptions on the focality or smoothness (spread) of the solution based on the experimental paradigm (Schmidt et al., 1999).

While such assumptions are reasonable, computational constraints impose artificial assumptions like limiting the number of sources or reducing the number of compartments in the head model. The MNE software (Gramfort et al., 2013) is one of the most widely used freely available source reconstruction packages. Source estimation on the MNE software package is based on the potential-based BEM formulation of the MEG forward model (Geselowitz, 1967, 1970; Mosher et al., 1999; Nuñez Ponasso, 2024); because of this, the forward system matrix is dense and the method is often limited to 3 compartments: the skin, skull, and pial surface. Furthermore, it is often limited to ~ 20,000 possible sources (Stenroos et al., 2014) even though using denser source spaces is possible with additional computational cost. Unfortunately, these limitations are present in most source-reconstruction software like MNE (Gramfort et al., 2013, 2014), FieldTrip (Oostenveld et al., 2010), Brainstorm (Tadel et al., 2011), or SPM (Friston et al., 2025); see the recent review in Feng et al. (2025). As a general principle, we should remove such limitations and base our inverse model on forward solutions that model the underlying bioelectromagnetics as accurately as possible (Dale and Sereno, 1993).

The main ingredient tying the forward model and the inverse model is the so-called *gain*, or *leadfield matrix* (LFM). If we assume that the orientations of all sources is fixed, then the LFM is a matrix of size (*Μ* × *N*), where *Μ* is the number of sensors and *N* is the number of sources —in all practical applications, *N* ≫ *M*. Each column of the leadfield matrix represents the vector of measurements calculated from the *N*th source in isolation, i.e. assuming that no other sources are activated. The major computational limitation to the number of sources is that, in the classical direct approach, the columns of the matrix must be computed one at a time.

The *charge-based boundary element fast multipole method* (or BEM-FMM) (Makarov et al., 2018, 2021) is a novel technique which employs the fast multipole method (Greengard et al., 2009) together with the charge-based formulation of the quasi-static bioelectromagnetic forward problem (Barnard et al., 1967; Makarov et al., 2018; Nuñez Ponasso, 2024) to compute forward solutions for a wide range of computational neuroscience models. This technique noted for its speed, accuracy, and ability to deal with high-resolution meshes with non-nested topology. In Makarov et al. (2020), a BEM-FMM toolkit to solve *transcranial magnetic stimulation* (TMS) forward problems was proposed. Due to *Lorentz’s reciprocity formula* (Heller and van Hulsteyn, 1992), the forward MEG problem can be solved by means of the forward TMS problem rather than computing forward solutions for equivalent current dipoles. Hence, we solve a TMS forward solution where the sensor coils act as TMS coils. We use Lorentz’s formula to “fill” the leadfield matrix by its rows instead of by its columns, which is the *reciprocal approach*. The advantage is clear: while the number of sources (columns) can be in the millions, the number of sensors (rows) is no larger than a few hundred in a typical MEG system. Furthermore, the coupling between BEM-FMM and the reciprocal MEG computation has two additional advantages: (1) it is sufficient to compute the charge densities induced by the sensors as TMS coils; and (2) in the TMS forward problem no singularities are present near the computational meshes, so *adaptive mesh refinement* (AMR) (Weise et al., 2022; Wartman et al., 2024) is not required for the accurate calculation of TMS solutions. This is in contrast to the direct computation of high-resolution EEG or MEG, which often requires AMR to improve numerical stability (Nuñez Ponasso et al., 2024; Wartman et al., 2025), and adds to the computational cost. The reader should note that the relationship between the TMS and MEG forward problems through reciprocity is established purely from a modeling point of view, and that no TMS sessions need to be performed on the participants in order to carry our methodology.

Once computed, the gain matrix is used to construct a linear inverse operator (see Section 2.8), which is then used to perform source localizations based on the classical minimum norm estimates (MNE) —not to be confused with the homonymous MNE-Software. Most importantly, the process of generating the MNE linear inverse operator from the gain matrix is independent of the process of generating the gain matrix itself. Therefore, we can test the performance of differently generated gain matrices using identical inverse methods. We emphasize that, in this work, we do not introduce new inverse methodology and instead focus on the forward methods.

Here, we replicated one-to-one the implementation of the inverse solvers employed by the MNE-Python Software, and compared our reciprocally-generated gain matrix against MNE-Python’s direct BEM models (1- and 3-layers) and a direct 3-layer isotropic FEM forward model.

We evaluated the performance of the inverse models derived from our high-resolution gain matrices by localizing *somatosensory evoked fields* (SEFs) elicited by right-hand median nerve stimulation. Results were compared with the standard MNE pipeline using those same BEM and FEM models. SEFs are known to be generated in the Brodmann area 3b (Purves et al., 2004; Brodmann, 2005) in the primary somatosensory cortex situated in the posterior wall of the central sulcus (Nuwer, 1998), making them an effective test case. Moreover, the localization of evoked somatosensory fields is relevant in clinical applications (Lüders et al., 1986). For simulated data, we generated MEG signals using direct (non-reciprocal) BEM-FMM with *b*-refinement (Wartman et al., 2024) to avoid bias from using the same forward operator. We assessed localization accuracy by comparing distances from the peaks and centroids of estimated activation regions to the ground-truth source dipole location at various noise levels.

## 2. Materials and methods

### 2.1. Reciprocal construction of the MEG leadfield matrix

The Lorentz reciprocity theorem (Supplement A) establishes a relation between two formally independent fields operating at the same frequency α, the field of a given dipole and the field of an arbitrary MEG sensor (magnetometer or gradiometer) operating as an induction (e.g. TMS) coil. In Supplement A it is shown that if both fields are assumed to be in phase, the reciprocity relation can be written in the following real form (without using complex phasors):

(1) $$p_{1}\cdot E_{2}^{r}(r_{1})=I_{0}\int_{s}B_{1}(s)\cdot n(s)ds.$$

Here, indices with value 1 refer to the current dipole, and indices with value 2 refer to the MEG sensor coil. Our notations are as follows:

- $p_{1}[A\;m]$ is the vector dipole moment of the primary current dipole source located at $r_{1}$ with current strength $J_{1}(r,r)=p_{1}\delta (r-\left.r_{1}\right)cos\;\omega t$.
- $I_{0}[A]$ is the amplitude of a harmonic coil current $I(t)=I_{0}\;cos(\omega t)$ flowing through the MEG sensor coil.
- $B_{1}[T]$ is the spatial distribution of the magnetic flux density $B_{1}(r;t)=B_{1}\left(r\right)\cos\left(\omega t\right)$ generated by the current dipole. The integral term is the magnetic flux through the surface *S* bound by the MEG coil.
- $E_{2}^{r}\left[V\;{s\;m}^{-1}\right]$ is the spatial distribution of the out-of-phase total electric field $E_{2}(r;t)=\omega E_{2}^{r}(r)sin\;\omega t$ generated by the MEG sensor operating as an induction coil, up to a multiplicative factor $\omega \left[s^{-1}\right]$.

The frequency ω can be chosen arbitrarily. Eq. (1) coincides with Equation (1) of (Nummenmaa et al., 2013) when the harmonic coil current is selected as stated above.

### 2.2. Multiple dipoles and one MEG sensor

It is shown in Plonsey (1972) that the quasi-static approximation to Maxwell’s equations can be used for EEG and MEG modeling. Eq. (1) can then be generalized to multiple dipoles and multiple MEG sensors using linearity. We consider multiple dipoles $p_{j},\;j=1,\ldots ,N$. We may assume that the dipole moments are perpendicular to the cortex (Hämäläinen et al., 1993), i.e. $p_{j}=p_{j}n_{j}$, where $n_{j}$ is the unit normal vector to the cortical surface at $r_{j}$. These dipoles generate the net static magnetic field $B(r)=B_{1}(r)+\cdots +B_{N}(r)$ obtained by superposition.

Applying Eq. (1) to each dipole individually, and summing, yields:

(2) $$\sum_{j=1}^{N}n_{j}\cdot E_{2}(r_{j})p_{j}=I_{0}\int_{S}n\cdot B(s)ds.$$

### 2.3. Multiple dipoles and multiple MEG sensors

We turn on only one *i*th induction coil (one sensor) at a time while leaving all others turned off. We assume the same current amplitude $I_{0}$ for every coil (sensor). For *M* sensors, this will give us *M* equations like Eq. (2) by running over all sensors (coils) when i=1,…,M. These equations can be written in compact matrix form (omitting indices 1 and 2 previously used):

(3) $$\left[\begin{array}{cccc} L_{11} & L_{12} & \ldots & L_{1N}\\ L_{21} & L_{22} & \ldots & L_{2N}\\ \vdots & \vdots & \ddots & \vdots \\ L_{M1} & L_{M2} & \ldots & L_{MN} \end{array}\right]\times \left[\begin{array}{c} p_{1}\\ p_{2}\\ \vdots \\ p_{M} \end{array}\right]=\left[\begin{array}{c} b_{1}\\ b_{2}\\ \vdots \\ b_{M} \end{array}\right];\;L_{ij}=n_{j}\cdot E_{i}\left(r_{j}\right);\;b_{i}=I_{0}\int_{S_{i}}n_{i}\cdot B(s)ds.$$

The matrix on the left-hand side of Eq. (3) is denoted by $\overset{ˆ}{L}$. This is indeed the standard unique MEG leadfield matrix which relates unknown dipole strengths $p_{j},\;j=1,\ldots ,N$ to the measured sensor responses $b_{i},i=1,\ldots ,M$ — a discretized representation of the forward problem (Knösche and Haueisen, 2022). The factor $I_{0}$ in Eq. (3) is superfluous; it can be chosen to be 1 A s^−1^. However, in contrast to the classical leadfield matrix definition (Knösche and Haueisen, 2022), the present formula explicitly specifies elements $L_{ij}$ of the leadfield matrix in terms of the reciprocal fields $E_{i}$ as, for example, in Nummenmaa et al. (2013) and Nolte (2003).

### 2.4. Comparison of direct and reciprocal approaches to constructing the lead field matrix

A direct approach widely used today in the boundary element method (BEM) software packages such as MNE (Gramfort et al., 2013), Brainstorm (Tadel et al., 2011), and FieldTrip (Oostenveld et al., 2010) does not employ electric fields of the MEG coils. Instead, magnetic fields of every dipole, $B_{1},B_{2},\ldots ,B_{N}$, which are present on the right-hand side of Eq. (3), are computed directly at all sensor locations for unit-strength dipoles at all putative source locations. In other words, $\overset{ˆ}{L}$ is filled *column-wise*, for every dipole separately. The number of such computations is equal to the number of dipoles *N*. We note that N≫M.

A currently less used alternative approach is a “reciprocal” BEM (Nummenmaa et al., 2013; Wendel et al., 2009; Laarne et al., 2000), where the lead field matrix is defined in terms of the reciprocal fields $E_{i}$. In other words, $\overset{ˆ}{L}$ is filled *row-wise*. We excite a single sensor at a time, compute the corresponding induced field everywhere in the cortex, e.g. at all dipole positions $r_{1},r_{2},\ldots ,r_{N}$, find the dot product of this field with the normal cortical vectors $m_{1},m_{2},\ldots ,m_{N}$, and finally obtain the complete *i*th row of $\overset{ˆ}{L}$. The number of such computations is equal to the number of sensors *M*.

### 2.5. Expansion of a cortical dipole density into global cortical basis functions

**Fig. 1.**
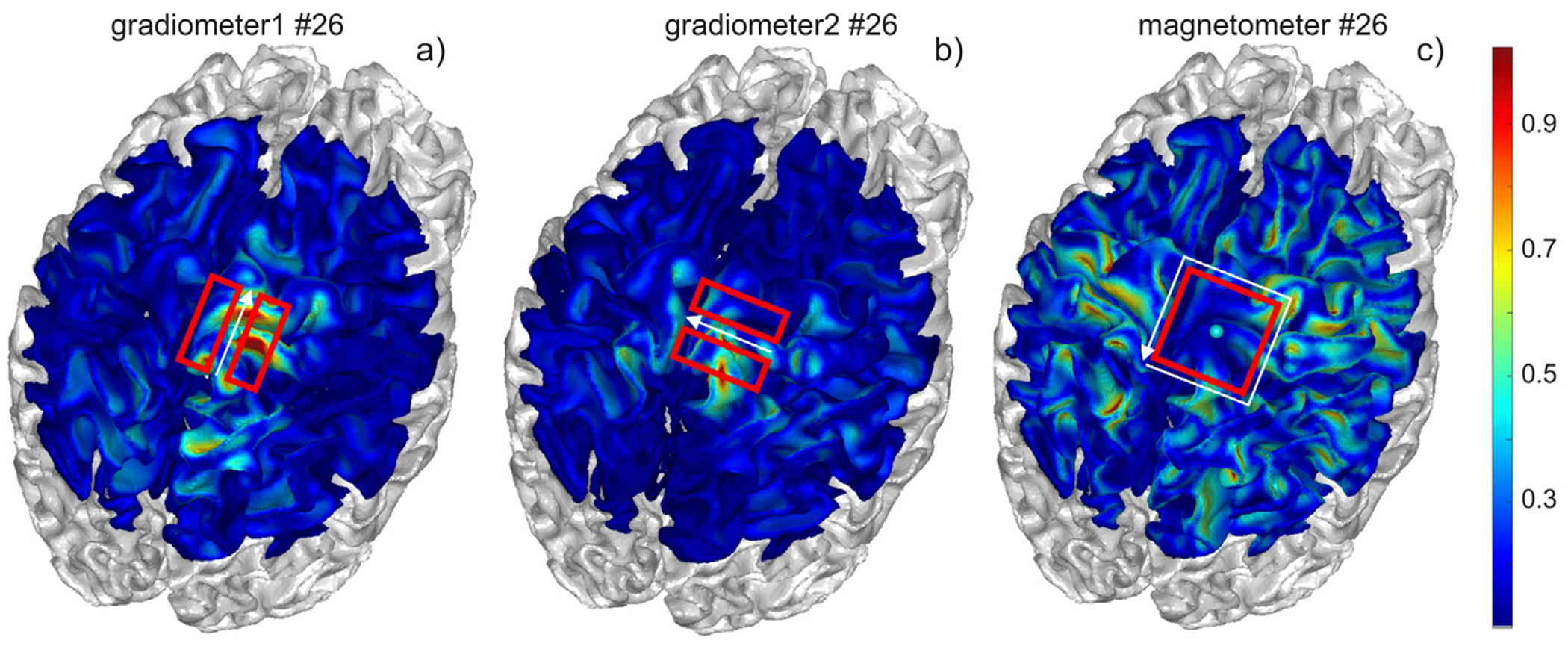
Normalized basis functions $L_{i}(r)$ for two perpendicular gradiometers and one magnetometer, respectively, computed for the first subject of a cohort described further in the text. The dominant current direction in the TMS mode is shown by the white arrow. The cortical surface chosen is the surface just outside white matter. The gradiometer basis functions are well localized over the cortical surface where the magnetometer basis functions are not.

The reciprocal approach for a dense source space can be interpreted as an expansion of the unknown continuous dipole density p(r) into a set of global basis functions, defined over the entire cortical surface, similar to spatial Fourier harmonics. To within a normalization constant, every such basis function is simply an electric field $E_{i}^{r}(r)$ of an MEG sensor (gradiometer or magnetometer) operating as a TMS coil and computed at a cortical surface of interest in Eq. (3). For the normal cortical dipole density, it is given by the projection of the electric field onto the normal cortical direction, $L_{i}(r)=n(r)\cdot E_{i}^{r}(r)$ where **r** belongs to the cortical surface of interest. Thus, we have

(4) $$p(r)=\sum_{i=1}^{K}\alpha_{i}L_{i}(r),$$

with unknown coefficients $\alpha_{i}$. The expansion in Eq. (4) is performed separately for two sets of perpendicular gradiometers and one set of magnetometers. The total number of unknown coefficients is therefore the total number of sensors. Fig. 1 illustrates the (normalized) basis functions $L_{i}(r)$ for two gradiometers and one magnetometer, respectively, computed for the first subject of a cohort described further in the text. The cortical surface chosen is the surface just outside the white matter resulting from a small shift of the white matter surface in the direction towards the gray matter surface.

### 2.6. Accelerated computation of normal component of E-field via charge densities

Since BEM-FMM computes surfaces charges, we can obtain the normal component of the total electric field without computing the secondary field (Makarov et al., 2020). Let *c* be the surface charge density and let **r** be a point at the WM-GM interface, then we have:

(5) $$n(r)\cdot E_{i}^{r}(r)=\frac{\sigma_{\text{WM}}}{\sigma_{\text{WM}}-\sigma_{\text{GM}}}c(r),\;\text{just outside the white matter surface}$$

Similarly, if **r** is a point at the GM-CSF interface, then we have

(6) $$n(r)\cdot E_{i}^{r}(r)=\frac{\sigma_{\text{CSF}}}{\sigma_{\text{GM}}-\sigma_{\text{CSF}}}c(r),\;\text{just inside the gray matter surface}.$$

This is in contrast to the finite element method (FEM) or the surface potential formulation of the BEM, where the calculation of the secondary field is necessary. In the following, we will choose the cortical surface as the one just outside the white matter interface and then use Eq. (5) directly.

### 2.7. Forward TMS computations using the FMM-accelerated charge-based boundary element method

To compute the forward TMS solution, we apply the charge-base boundary element fast multipole method (Greengard et al., 2009; Makarov et al., 2018). This method uses the charge-based formulation of the boundary element method, where the charge density ρ induced by an arbitrary incident field $E^{i}$ is computed according to the following integral equation (Makarov et al., 2018; Nuñez Ponasso, 2024):

(7) $$\frac{\rho \left(r\right)}{2\varepsilon_{0}}-K(r)n(r)\cdot \int_{S}\frac{\rho \left(r^{\prime }\right)}{4\pi \varepsilon_{0}}\frac{r-r^{\prime }}{{\left|r-r^{\prime }\right|}^{3}}dS\left(r^{\prime }\right)=K(r)E^{i}(r)\cdot n(r)\;\text{for all}\;r\in S.$$

Here, we are assuming that the conductivity σ is isotropic, and that: *S* is the surface of discontinuity of σ. The vector: **n**(**r**) is the outward normal to the surface *S* at a point: **r** of: *S*. The constant $\varepsilon_{0}\approx 8.8541\;\times \;10^{-12}\left[F\;m^{-1}\right]$ is the permittivity of free space. The dimensionless scalar: K(r) is the *conductivity contrast* at an interface point **r** of *S*, defined as

(8) $$K(r)=\frac{\sigma_{-}-\sigma_{+}}{\sigma_{-}+\sigma_{+}},$$

where $\sigma_{+}\left(\sigma_{-}\right)$ is the conductivity just outside (just inside) of the surface *S* at the point **r**.

In our case, the incident electric field is the one generated by a current loop of unit strength following the outline of the MEG sensors; this is equivalent to calculating a TMS solution using the sensors as stimulation coils. To model the incident field $E^{i}$ generated by a TMS coil, we use a series of current elements (Makarov et al., 2020) of current $i_{j}(t)$ with orientation vector $s_{j}$ and center $p_{j}$, for j=1,…,ℓ. These current elements can be distributed uniformly across the cross-section of the coil to model Litz wires, or non-uniformly for wires susceptible to the skin effect. The primary field $E_{j}^{i}$ produced by the *j*th current element is given by (Balanis, 2012; Makarov et al., 2020),

(9) $$E_{j}^{i}(r)=-\frac{\partial i_{j}(t)}{\partial t}\frac{\mu_{0}}{4\pi }\frac{s_{j}}{\left|r-p_{j}\right|}.$$

Where $\mu_{0}\approx 1.2566\;\times \;10^{-6}\;N\;A^{-2}$ is the permeability of free space. The primary field is calculated by aggregating the contribution of each current element:

(10) $$E^{i}\left(r\right)=\sum_{j=1}^{\ell }E_{j}^{i}\left(r\right)=-\sum_{j=1}^{\ell }\frac{\partial i_{j}\left(t\right)}{\partial t}\frac{\mu_{0}}{4pi}\frac{s_{j}}{\left|r-p_{j}\right|},$$

and this summation term can be accelerated using the fast multipole method (FMM) (Greengard et al., 2009).

Using the Galerkin method, the charge-based Eq. (7) becomes discretized as:

(11) $$\rho_{n}=2\varepsilon_{0}K\left(r_{n}\right)n_{n}\cdot \left(\sum_{\begin{array}{c} m=1\\ m\neq n \end{array}}^{N}\frac{A_{m}\rho_{m}\left(r_{n}-r_{m}\right)}{4\pi \varepsilon_{0}{\left|r_{n}-r_{m}\right|}^{3}}+E^{i}\left(r_{n}\right)\right).$$

The solution of Eq. (11) can be accelerated by using FMM to compute the summation term. Using the first-order approximation $\rho_{n}^{0}=E^{i}r_{n}$, Eq. (11) can be used to iteratively solve for $\rho_{n}$ (Nuñez Ponasso, 2024): At every step k — having computed the successive approximations $\rho_{n}^{\left(0\right)},\rho_{n}^{\left(1\right)},\ldots ,\rho_{n}^{\left(k\right)}$ — we compute a *generalized minimum residual* (GMRES) solution by calculating the parameters $\alpha_{0},\alpha_{1},\ldots ,\alpha_{k}$ minimizing the residual in the expression

(12) $$\rho_{n}=\alpha_{0}\rho_{n}^{\left(0\right)}+\alpha_{1}\rho_{n}^{\left(1\right)}+\cdots +\alpha_{k}\rho_{n}^{\left(k\right)}.$$

Following this procedure, the charge density ρ is computed on all interface tissues, and in particular on the entire WM-GM interface. More generally, if we want to allow a degree of deviation from the normal direction, we would need to compute the total E-field on the white matter mesh. In this case, the computation of the forward solution can be accelerated by restricting the *region of interest* (ROI), i.e. the region where the total E-field is computed, to a subset of the white matter surface. We carried simulations where we restricted to the subregion of the WM-GM interface which lies within 7 cm of each sensor centroid. By the inverse-squared decay of the magnitude of the E-field, this radius was observed to be sufficient to approximate the E-field away from this distance as zero. More information on the parameters for the BEM-FMM solution using GMRES is included in Table 1.

### 2.8. Formulation and solution of the inverse problem

We would like to compare high-resolution inverse models derived from our reciprocal solver against the widespread and well-established inverse solutions generated using MNE’s direct BEM solvers (Gramfort et al., 2013). To this end, we follow one-to-one the inverse methodology implemented in MNE-Python (Gramfort et al., 2014), these methods are discussed in detail on MNE-Python’s online documentation. By doing so, we aim to eliminate any possible discrepancies due to the use of different inverse methods and ensure that different results are solely caused by the use of different forward modeling techniques.

We find the solution of the inverse problem as a *maximum a posteriori* solution, where given a set of prior assumptions (or distributions) on the source space, the noise, and the physical model, we find the source strength distribution of maximum likelihood —this is discussed in the Bayesian framework of Knösche and Haueisen (2022), and it is the framework used by MNE-Python (Gramfort et al., 2014, 2013). The inverse problem is then formulated using three main ingredients:

- The *M* × *N* leadfield matrix *L* —described in Eq. (3) — which is a *modeling* term describing a linear relationship between source strengths and MEG sensor measurements;
- the source covariance matrix *R* (or source weighting matrix), which is a Bayesian *prior* term, and can be seen as an initial assumption on the properties of the source space.
- the noise covariance matrix Σ, which is an *empirical* term capturing spatiotemporal correlations of the sensor readings.

Within the leadfield matrix, we implicitly assume that the sources are constrained to the cortex, with orientation normal to the cortical surface.

In its simplest form (without source priors or noise covariance matrices), the inverse problem is formulated as a least-squares minimum norm problem (Hämäläinen and Ilmoniemi, 1994), which, for an underdetermined problem (i.e., when *M* < *N* as is our case), can be posed as:

(13) $$\text{min}\;\|x{\|}^{2}=x_{1}^{2}+x_{2}^{2}+\cdots +x_{N}^{2}\;\text{s.t.}\;Lx=b.$$

This problem has a unique solution, and the solution **x** is given explicitly by means of the pseudoinverse of *L*:

(14) $$x=L^{+}b=L^{\text{⊺}}{\left(LL^{\text{⊺}}\right)}^{\left(-1\right)}b.$$

The pseudoinverse $L^{+}=L^{⊺}{\left(LL^{⊺}\right)}^{-1}$ is called the *inverse operator* in this case. In general, if we want to introduce the source and noise covariance matrices, we use the following *regularized inverse operator* (Tarantola, 2005; Hämäläinen and Ilmoniemi, 1994; Dale and Sereno, 1993):

(15) $$M=RL^{⊺}{\left(LRL^{⊺}+\lambda^{2}\Sigma \right)}^{-1},$$

where λ is a regularization parameter. Note that with *R* = *I* and λ=0 we recover the usual pseudoinverse. The inverse solution **x** is computed as x=Mb. For a derivation of this regularized pseudoinverse operator, see Supplement B.

### 2.9. Depth weighting: Leadfield matrix column normalization

The sensitivity of MEG sensors to a single dipolar source depends highly on the location of the source (Hillebrand and Barnes, 2002): Deeper sources produce weaker magnetic fields at the sensor positions —as predicted by the Law of Biot and Savart (Griffiths, 1999)— and MEG sensors are less sensitive to the radially-oriented sources at the crests of the gyri (although these constitute a smaller fraction of the total cortical surface). Dipoles with weaker magnetic fields produce columns in the leadfield matrix *L* with a small norm; this will ultimately cause a bias towards superficial sources in the minimum norm solution.

In order to reduce this bias, we can normalize the columns of the leadfield matrix, (Fuchs et al., 1999; Lin et al., 2006). Normalizing the columns of the leadfield matrix corresponds to selecting the following source-covariance matrix:

(16) $$R=\text{diag}\left(w_{1}^{-1/2},w_{2}^{-1/2},\ldots ,w_{N}^{-1/2}\right),$$

where

(17) $$w_{j}=\left\|\left(L_{1,j},L_{2,j},\ldots ,L_{M,j}\right)\right\|;\;j=1,\ldots ,N,$$

is the norm of the *j*th column of the leadfield matrix *L*. In the MNE-Python software, we obtain this normalization by requiring the dipole orientations to be normal to the cortical surface and by setting the depth-weighting parameter *p* = 1 (Lin et al., 2006).

We also applied data whitening (Dale and Sereno, 1993), which involves a transformation of the leadfield matrix given by $\overset{‾}{L}=\Sigma^{-1/2}L$, and the corresponding transformed inverse operator becomes

(18) $$\bar{M}=M\;\Sigma^{1/2}=R{\bar{L}}^{⊺}{\left(\bar{L}R{\bar{L}}^{⊺}+\lambda^{2}I\right)}^{-1}.$$

### 2.10. Dynamic statistical parametric mapping (dSPM)

We can modify minimum norm estimates by employing *dynamic statistical parametric mapping (dSPM)* (Dale et al., 2000). This method utilizes a noise normalized post-hoc weighting by computing

(19) $$R^{\text{*}}=M\text{Σ}M^{⊺}.$$

The inverse solution **x** is then normalized by

(20) $$x^{*}\frac{x^{⊺}x}{\text{trace}(R^{*})}.$$

$R^{*}$ is effectively the variance of the dipole strengths given a fixed dipole orientation. Therefore, if the noise covariance is computed over a sufficiently long time interval, $x^{*}$ is analogous to a statistical z-score (Dale et al., 2000). That is, dSPM gives a normalized inverse solution in which the most statistically relevant dipole strengths are found among the highest percentiles. In general, the dipole strengths can vary greatly along regions of high curvature variation, i.e. along transitions from surface crests to sulci. Thus normalizing by the diagonal elements of $R^{*}$ will bias the most statistically relevant solutions towards regions of least variation. Since **x** is found such that superficial sources along the surface crests are prior corrected, $x^{*}$ will yield strong sources predominately in the surface sulci.

### 2.11. Evoked MEG responses to median nerve stimulation

#### Subjects, stimuli, and task.

Five healthy adults participated in the recordings at the Athinoula A. Martinos Center for Biomedical Imaging, Massachusetts General Hospital (MGH), Boston, MA, between years 2004 and 2005. The study protocol was approved by the MGH Institutional Review Board (protocol #1999P010946). The subjects gave written informed consent prior to participation. The subject age range was 25–40 (mean 31 years, 2 females). Here, the subjects will be referred to by the subject codesMGH01–MGH05.

#### MRI acquisition and analysis.

T1-weighted structural MRIs of the head were recorded with a magnetization-prepared rapid gradient echo (MPRAGE) sequence at 1.33 mm spatial resolution (Siemens Avanto, Trio, or Sonata; Siemens Medical Solutions, Erlangen, Germany). The MRI data were segmented using automated software and the results were used for constructing the boundary element models necessary for source modeling. For source estimation with BEM-FMM, the MRI data were segmented using the headreco pipeline of SimNIBS (Nielsen et al., 2018). For source estimation using MNE the MRI data were segmented and 3-layer BEMs (scalp, outer skull, inner skull) built using the Freesurfer software (Reuter et al., 2012).

#### MEG recordings and sensor-space analysis.

Whole-head 306-channel MEG (VectorView; MEGIN Oy, Finland) data were recorded in a magnetically shielded room (Cohen et al., 2002). The instrument employs sensor triplets (one magnetometer and two planar gradiometers) at 102 measurement locations. Simultaneously, 70-channel EEG (not analyzed here) as well as horizontal and vertical electro-oculogram (EOG) were recorded. All signals were bandpass-filtered to 0.01–250 Hz and sampled at 1 kHz.

At the start of the MEG session, the locations of 4 head position indicator (HPI) coils attached to the scalp and several additional scalp surface points were recorded with respect to fiduciary landmarks (nasion and two preauricular points) using a 3-D digitizer (Fastrak Polhemus, VT, USA). During MRI–MEG coordinate system alignment, the fiduciary points were then identified from the structural MRIs. Using the HPI and scalp surface locations, this initial approximation was refined using an iterative closest-point search algorithm.

During the MEG recordings, the subjects received 0.2 ms square wave pulses at a suprathreshold intensity at the right wrist over the median nerve (Telefactor S88, Grass Instrument Company, Quincy, MA, USA). The stimuli were presented at a pseudorandom interstimulus interval of 3–9 s. The task was to respond to each stimulus by lifting the left hand index finger as quickly as possible.

**Fig. 2.**
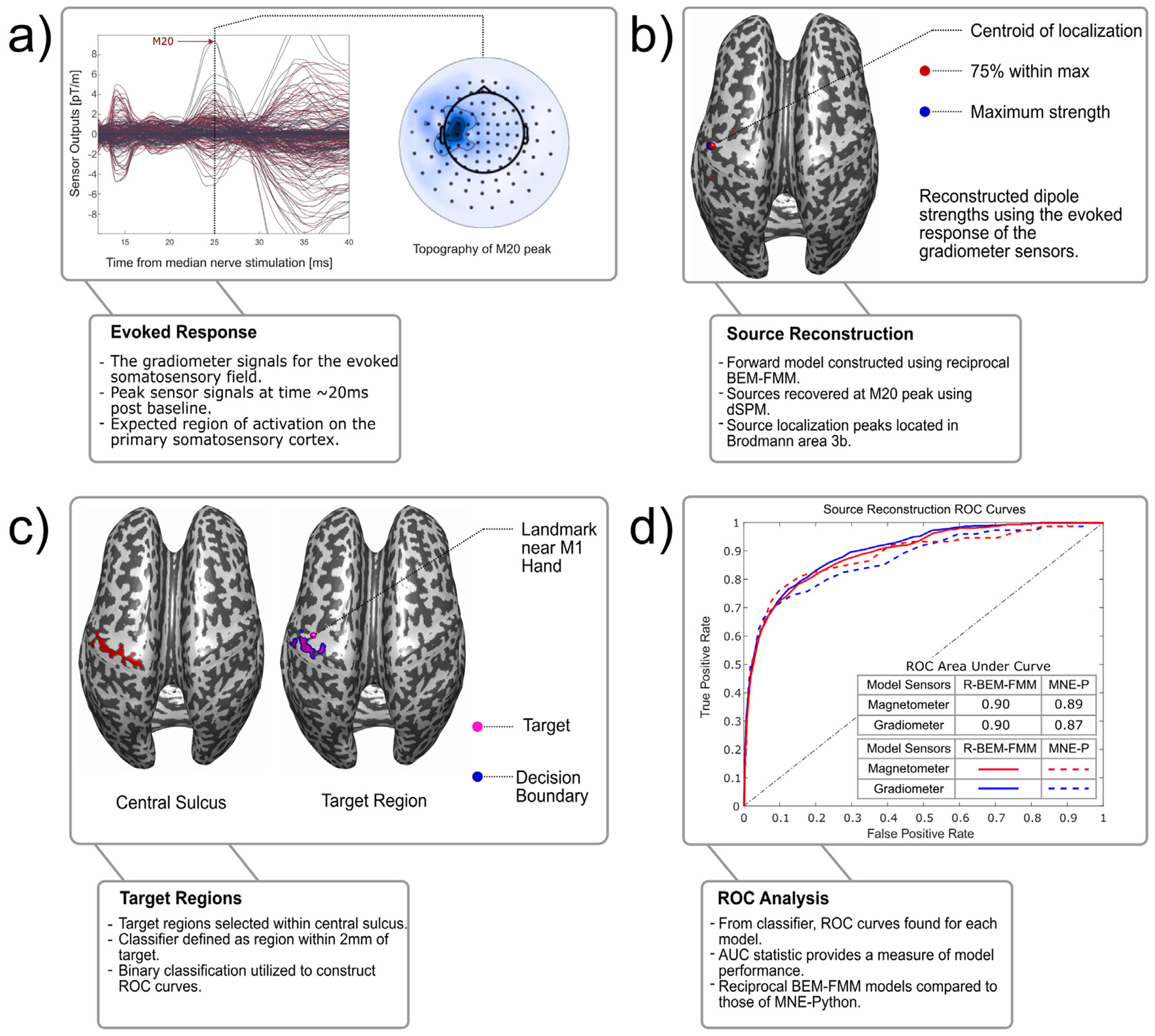
The workflow of the source localization ROC analysis, using subject MGH01. (a) The gradiometer signals for the evoked somatosensory field, along with their corresponding topographies. The M20 peak occurs at a latency of approximately 20 ms post stimulation. (b) The Reciprocal BEM-FMM inverse model dSPM solution for M20 peak plotted on the inflated white matter. The centroid of 75% strength threshold is plotted as a red sphere, and the maximum strength is plotted as a blue sphere. (c) Plot of the tagged central sulcus (left,red), the approximate M1 hand (right, magenta sphere), and the tagged true-positive region for binary classification (right, magenta) and *decision boundary* (right,blue), i.e. the boundary of the true-positive region. (d) The ROC curves for the reciprocal BEM-FMM (solid line) and MNE-Python (dashed line) inverse models. We compare models generated from gradiometer (blue) and magnetometer (red) data. AUC values for these models can be found in Table 3.

Evoked responses were averaged with respect to the median nerve stimuli in a time window from 200 ms prestimulus to 1150 ms post-stimulus with a band-pass of 0.3–1000 kHz. Epochs containing EOG signals exceeding 150 mV peak-to-peak amplitude were automatically discarded from the averages. After rejecting trials containing artifacts, the evoked responses contained 79 ± 19 (mean ± standard deviation) trials. The noise covariance matrix was estimated from a time window of −200 to −20 ms relative to the median nerve stimuli. Fig. 2A shows an example of the evoked gradiometer signals and sensor topographies for subject MGH01.

For the MNE-Python inverse models, the source space was generated with the setup_source_space procedure with ico5 source spacing, which produces 20477 ± 5 source dipoles evenly spread across the cortex. In our BEM-FMM inverse models, the source space consists of the triangle centers of the white matter mesh of each subject, which in unrefined headreco models comprise ~237,000 triangles. The full set of tissue layers, together with their conductivity values, average number of triangles, and relevant bibliographic references for both our BEM-FMM models and the MNE-Python models are included in Table 2, see also (SimNIBS Documentation, 2025b).

### 2.12. Visualization of the reconstructed dipole strength densities

We visualized the normalized reconstructed source strength density exceeding a specified threshold *T* relative to the maximum strength. Specifically, if $s_{\text{max}}$ represents the largest source strength magnitude, then we plot all sources with strength $s\geq T\cdot s_{\text{max}}$. Additionally, we plot the centroid (e.g. geometric mean) of the region with sources exceeding a given threshold. Fig. 2B shows an example of the centroid at a 75% threshold (as well as the source of maximum strength) of the reciprocal BEM-FMM dSPM solution for subject MGH01.

To compare the results of MNE-Python’s and BEM-FMM inverse models, we plotted the reconstructed dipole source density on the same inflated white matter mesh (Fischl et al., 1999) obtained from the subject headreco segmentations. Whenever coregistration was needed, we obtained optimal transformations using an *iterative closest point (ICP)* algorithm (Bergström and Edlund, 2014). The source strength densities obtained from MNE-Python are sparse relative to the mesh, and hence were smoothed iteratively to replicate the native visualization of MNE-Python. The smoothing is performed with a *k*-nearest neighbors algorithm at each triangle center, and averaging the neighbor values.

### 2.13. The statistical ROC metric

The *receiver operating characteristic (ROC)* curve is a tool to evaluate the performance of binary classification models (Knösche and Haueisen, 2022; Hastie et al., 2009). We treat source localization as a binary classification problem by specifying a target region for the “true” dipole sources. For the experimental data, the expected region of the “true” sources will lie in the central sulcus, with the precise location unknown. To avoid adding biases, we selected a sufficiently large subregion of the central sulcus closest to the M1-hand region, which we tagged manually. This is done computationally by (1) tagging the mesh triangles of the white matter that lie in central sulcus; and (2) choosing a subregion of the tagged central sulcus, consisting of 40% of the total central sulcus area closest to the M1-hand region. The rationale for this choice of 40% was that across all subjects we observed that this threshold included the entire posterior wall of the central sulcus lying immediately across the M1 hand region, as well as a small fraction of adjacent cortical regions. We note, however, that the general comparative performance does not heavily depend on the choice of percentage, see Supplement C.

The tagging of the central sulcus points is done by manually selecting points along the sulcus and then using a *k*-nearest neighbors search to locate the nearest mesh faces. Among this set of faces, we tag only those with a mean curvature value less than 1 (mean curvature *H* < 1 indicates the presence of a “valley” on the surface (do Carmo, 2016)). From a selected point in the M1-hand region, we chose the smallest radius *R* such that the area of the triangles with centers at distance < *R* from the M1-hand point is at least 40% of the total area of the central sulcus tagged. These flagged points near the M1-hand are our target region of true sources for binary classification. The central sulcus and target region tagged for the subject MGH01 are seen in Fig. 2C.

We classify sources as *true positives* (TP), *false negatives* (FN), *false positives* (FP), and *true negatives* (TN), based on the following procedure: begin with a given threshold percentage *T*; if a source dipole on the target region has a normalized source strength *s* > *T*, then the source dipole is considered a TP; similarly, if *s* < *T*, then it is a FN. Likewise, if a source dipole outside the target region has a normalized source strength *s* > *T*, then it is a FP; if *s* < *T*, then it is a TN.

With this binary classification, the ROC curve is generated by plotting the rate of false positives, *FP*/(*FP* + *TN*), versus the rate of true positives, *TP*/(*TP* + *FN*), as they vary across all threshold percentages *T* from 0 to 100%. The *area under the ROC curve (AUC)* serves as a numerical measure of the efficacy of the model: if *AUC* = 1, then the model is a perfect classifier; whereas if *AUC* ≤ 0.5, then the model behaves no better than a random binary classifier (Hastie et al., 2009). Example ROC curves for subject MGH01 are found in Fig. 2D.

### 2.14. Model evaluation with simulated ground truth data

#### Direct simulation of evoked somatosensory fields from equivalent current dipoles.

**Fig. 3.**
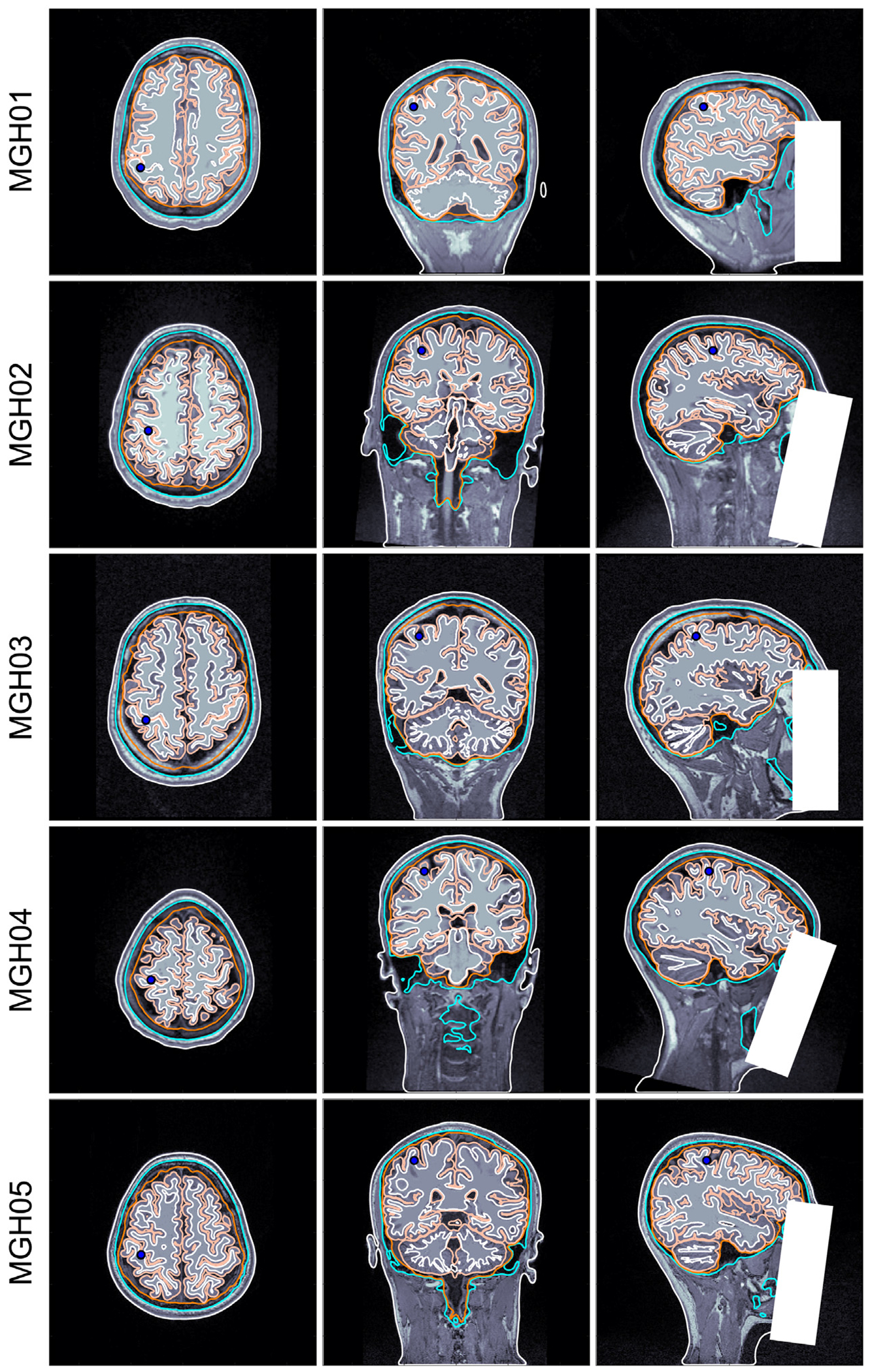
Placement of selected dipoles (blue circles) on Brodmann area 3b for each of the 5 subjects. Each tissue layer used in the model has the following color assignation: Skin —white (outer); Skull —cyan; CSF —orange; GM —apricot; WM —white (inner).

For each of the 5 subjects, we manually selected a location for a tangentially-oriented dipole on the posterior wall of the central sulcus (Brodmann area 3, see Fig. 3).

**Fig. 4.**
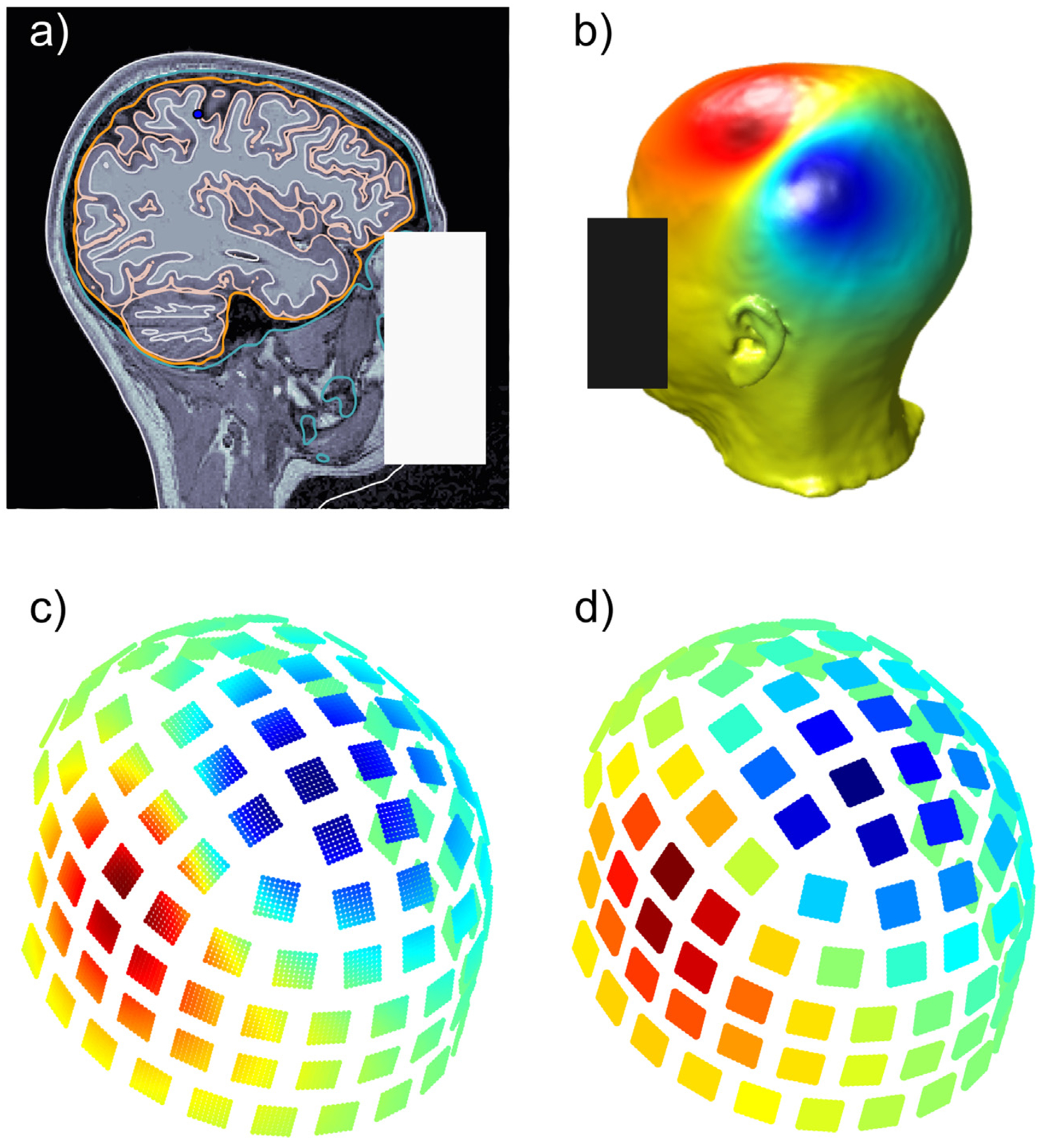
Workflow for the generation of simulated magnetometer data: (a) dipole placement in the primary somatosensory cortex for subject MGH05; (b) surface potential on the skin calculated BEM-FMM with *b*-refinement; (c) **B**-field calculated at 64 observation points for each magnetometer; (d) simulated sensor outputs by numerical computation of the integral of the normal component of **B** at the observation points.

The **B**-field corresponding to each selected dipole was generated using BEM-FMM with *b*-refinement (Makarov et al., 2021; Wartman et al., 2025), and sampled at 64 observation points for each magnetometer sensor, and 128 observation points for each gradiometer sensor. The sensor readings were then computed from the values of the flux **B** at the observation points by approximating the surface integral

(21) $$\int_{S}B(s)\cdot n(s)dS_{j}(s)$$

on the surface $S_{j}$ enclosed by sensor *j* (Knösche and Haueisen, 2022). In the case of magnetometers, this integral is approximated directly, see Fig. 4. In the case of gradiometer pairs, the normal vectors of each coil are taken in opposite directions and the integral is divided by the distance separating each coil. The simulated magnetometer and gradiometer signals were used for source reconstruction to evaluate the performance of our inverse model in the absence of noise.

#### Source localization of simulated noisy somatosensory evoked fields at various SNR levels.

We tested the stability of our inverse models by incorporating different levels of noise to our simulated MEG signals corresponding to the selected dipoles. The noise was modeled by randomly sampling 2000 dipoles across the entire cortical surface: each dipole was centered on the midsurface between the gray and white matter surfaces and oriented normally to the GM surface. For each of the randomly selected dipoles, we computed the forward BEM-FMM solution with *b*-AMR and created a direct leadfield matrix *L* of size 306 × 2000 by letting each column be the MEG sensor signals corresponding to each dipole. We generated 1000 samples of noisy signals at each target signal-to-noise ratio level with the following procedure:

1. For each ω=1,…,1000 we sampled a random 2000 × 1 vector of dipole strengths $X_{\omega }∼𝒢(0,\sigma )$ following a gaussian distribution of zero mean and standard deviation σ=1.
2. By the quasi-static Maxwell assumption, the noise $\epsilon_{\omega }$ is computed from the direct leadfield matrix as
(22) $$\epsilon_{\omega }=LX_{\omega },$$
  and it is then divided by its norm, so that $\epsilon_{\omega }$ is a unit vector.
3. The total signal was defined in terms of a parameter λ between 0 and 1 as
(23) $${\text{SIGNAL}}_{\omega }(\lambda )=(1-\lambda )\epsilon_{\omega }+\lambda b,$$
  where *b* is the normalized (unit vector) MEG signals of the selected dipole in the posterior wall of the central sulcus (described above). The signal is subsequently re-scaled to a realistic magnitude (Murakami and Okada, 2015).
4. The SNR is calculated according to the formula:
(24) $${\text{SNR}}_{\omega }(\lambda )=\sqrt{\frac{\text{Power}\left({\text{SIGNAL}}_{\omega }(\lambda )\right)}{\text{Power}\left((1-\lambda )\epsilon_{\omega }\right)}}=\frac{\text{std}\left((1-\lambda )\epsilon_{\omega }+\lambda b\right)}{\text{std}\left((1-\lambda )\epsilon_{\omega }\right)}.$$
  A value $\lambda^{*}$ between 0 and 1 was calculated numerically, so that
(25) $${\text{SNR}}_{\omega }\left(\lambda^{\text{*}}\right)={\text{SNR}}_{0}$$
  for each target signal-to-noise ratio ${SNR}_{0}$

The SNR values we tested were 81, 27, 3, 2, and 1.5. For each sample at every SNR level, we performed source localization and recorded the distance from the source dipole to (1) the reconstructed source of maximum strength (peak) and (2) to the centroid of the 75% activation threshold.

Both for the noiseless source localization, and the source localization at different SNR levels, we employed the minimum norm estimation described in Section 2.8, without the use of dSPM. The reason for the omission of the dSPM step is that this method requires the estimation of a noise covariance matrix, which for simulated data may result in the introduction of biases.

#### Error maps for source localization at various noise levels.

Using the forward BEM-FMM solution for each of the 2000 dipoles placed uniformly at random over the cortex —which we described above— we generated error maps indicating the average localization error at several *absolute noise levels* at each of the dipole locations. For each dipole, of index *j*, we created 500 samples of noise signals by multiplying the 306 × 2000 lead-field matrix *L* by a random vector of strengths $X_{\omega }$, where we impose the condition that $X_{\omega }(j)=0$, so that the noise does not contribute to the source signal. The total signal was computed for a *fixed* absolute noise percentage $0\leq \lambda_{0}\leq 1$ as

(26) $${\text{SIGNAL}}_{\omega }=\left(1-\lambda_{0}\right)Le_{j}+\lambda_{0}LX_{\omega },\;\text{for samples}\;\omega =1,\ldots ,500.$$

Here, $e_{j}$ is the vector with all entries equal to 0 except at the position *j* where it has the value 1. For each of the sample signals, we performed source reconstruction and measured the distance from the centroid of the estimated sources at the 75% threshold to the source dipole, as well as the distance from the peak of the estimated region of activation to the source. The reason for taking a fixed absolute noise level rather than a fixed SNR is that having a fixed SNR will force deep sources to have a much larger magnitude than the superficial ones. So using a fixed SNR limits the comparability of the quality of fits among different cortical regions and different sensor data. By keeping a fixed noise level, we avoid these issues.

## 3. Results

### Experimental data: evoked MEG responses to median nerve stimulation.

**Fig. 5.**
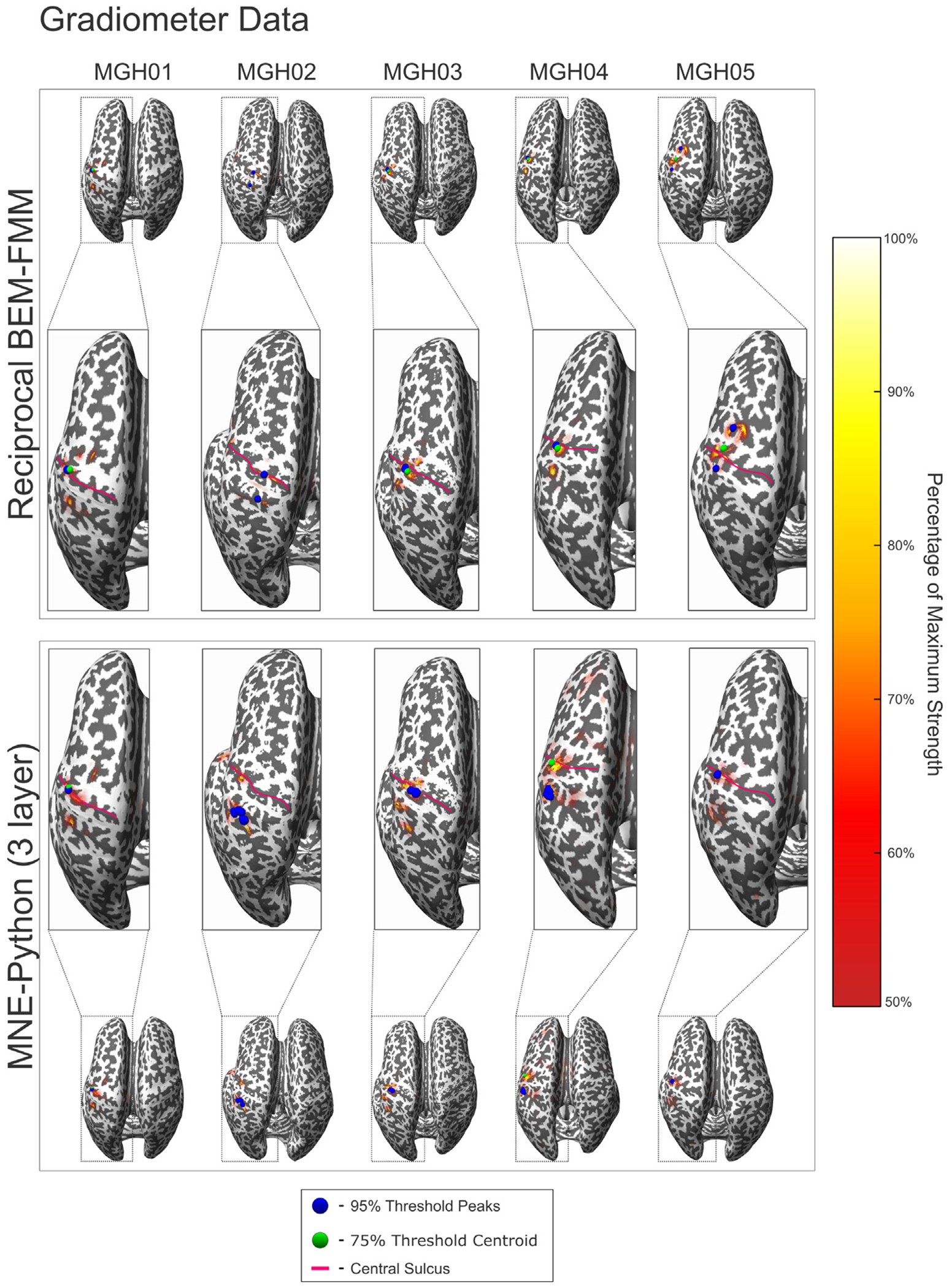
Reciprocal BEM-FMM inverse model and MNE-Python (3 layer) source localization results for all subjects using gradiometer data and dSPM. The blue spheres indicate sources with strengths within 95% of the maximum (peaks). The green sphere indicates the centroid of all localized sources with strengths within 75% of the maximum. The red curves denote the central sulcus of each subject. The colorbar denotes sources with strengths within 50%–100% of the maximum.

**Fig. 6.**
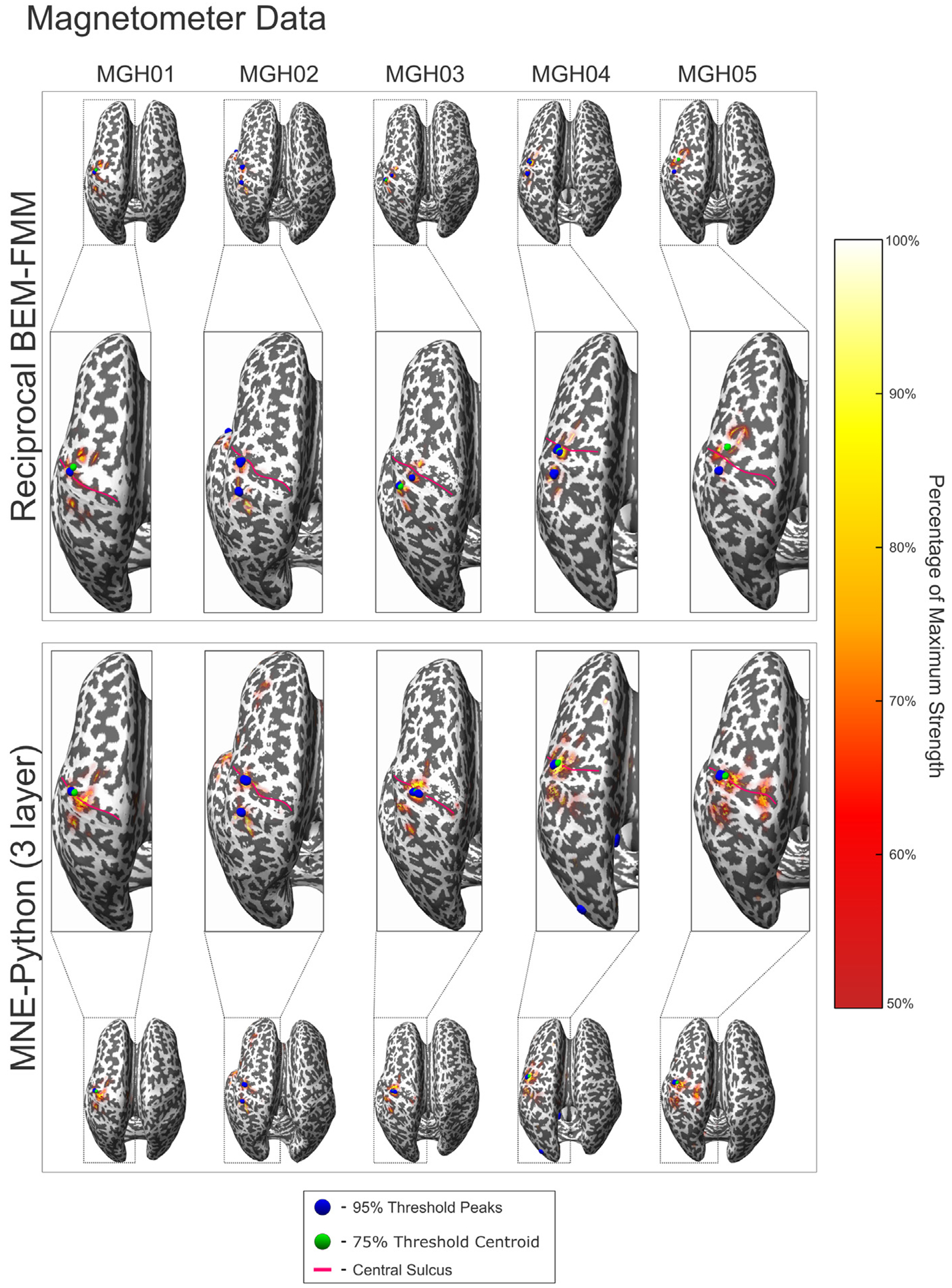
Reciprocal BEM-FMM inverse model and MNE-Python (3 layer) source localization results for all subjects using magnetometer data and dSPM. The blue spheres indicate sources with strengths within 95% of the maximum (peaks). The green sphere indicates the centroid of all localized sources with strengths within 75% of the maximum. The red curves denote the central sulcus of each subject. The colorbar denotes sources with strengths within 50%–100% of the maximum.

Figs. 5 and 6 show the source localization peaks, centroids and 50% threshold activation for the evoked somatosensory fields for each subject calculated using dSPM. Each figure shows the solutions computed using reciprocal BEM-FMM and MNE-Python based on a 3-layer BEM, with solutions in Fig. 5 computed from gradiometer data, and solutions in Fig. 6 computed from magnetometer data. We computed ROC curves from each of these models, which can be found in Supplement C. Table 3 shows the AUC values corresponding to each subject model using both reciprocal BEM-FMM, the 1- and 3-layer BEM models of MNE-Python, and a 3-layer anisotropic FEM model created using Brainstorm (Tadel et al., 2011): the FEM geometry was created from skin, inner skull and outer skull BEM-layers of headreco using the iso2mesh pipeline(Fang and Boas, 2009; Tran et al., 2020) and the forward model was computed from a sub-sampled headreco white matter mesh with ~ 20,000 dipoles and using the software DUNEuro (Schrader et al., 2021).

### Synthetic data: source reconstruction for simulated somatosensory fields at various noise levels.

**Fig. 7.**
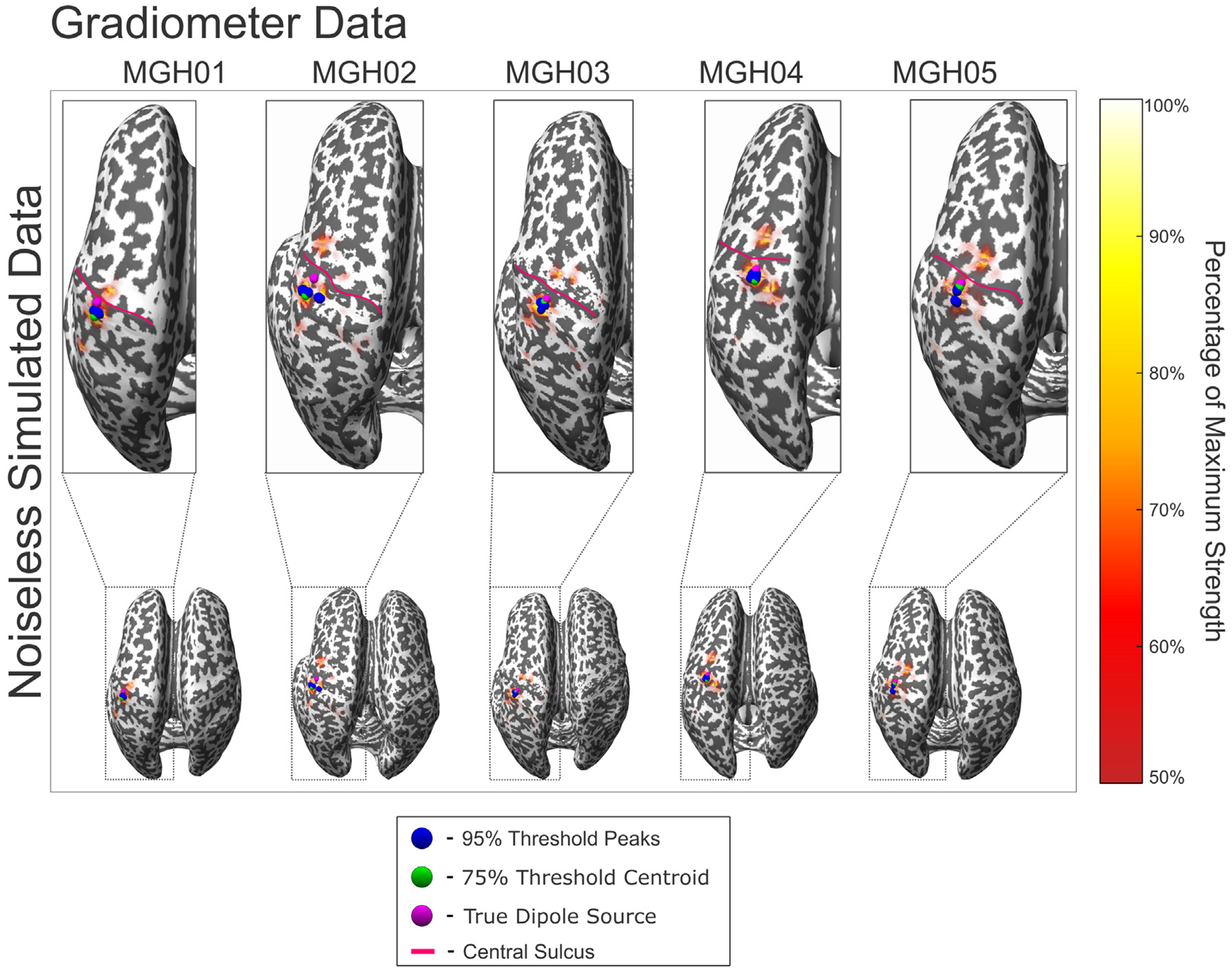
Reciprocal BEM-FMM source localization results for all subjects using synthetic gradiometer signals without noise. The magenta sphere indicates the source dipole used to generate the synthetic data. The blue spheres indicate sources with strengths within 95% of the maximum (peaks). The green sphere indicates the centroid of all localized sources with strengths within 75% of the maximum. The red curves denote the central sulcus of each subject. The colorbar denotes sources with strengths within 50%–100% of the maximum.

**Fig. 8.**
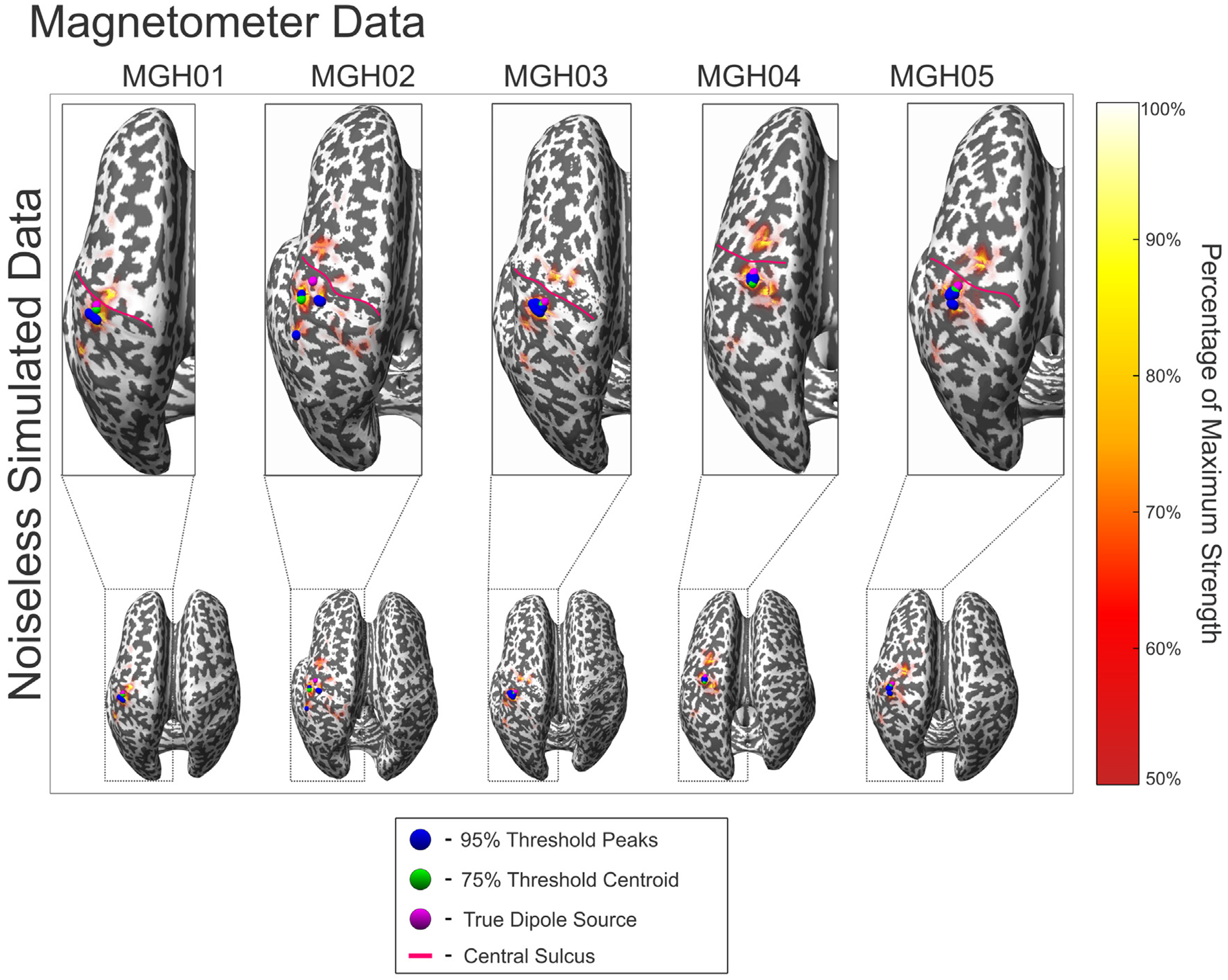
Reciprocal BEM-FMM source localization results for all subjects using synthetic noiseless magnetometer signals. The magenta sphere indicates the source dipole used to generate the synthetic data. The blue spheres indicate sources with strengths within 95% of the maximum (peaks). The green sphere indicates the centroid of all localized sources with strengths within 75% of the maximum. The red curves denote the central sulcus of each subject. The colorbar denotes sources with strengths within 50%–100% of the maximum.

Figs. 7 and 8 show the source localization peaks, centroids, and 50% threshold activation for the noiseless evoked somatosensory fields of each subject, where noiseless synthetic MEG signals were generated from the indicated source dipole. Tables 4a and 4b show the aggregated distance error statistics corresponding to the centroid-to-source and peak-to-source distances, respectively, from the ground truth source across varying levels of noise, using synthetic magnetometer and gradiometer data. Values in Table 4 are averaged across each subject; complete statistical data tables for individual subjects are shown in Supplement C.

**Fig. 9.**
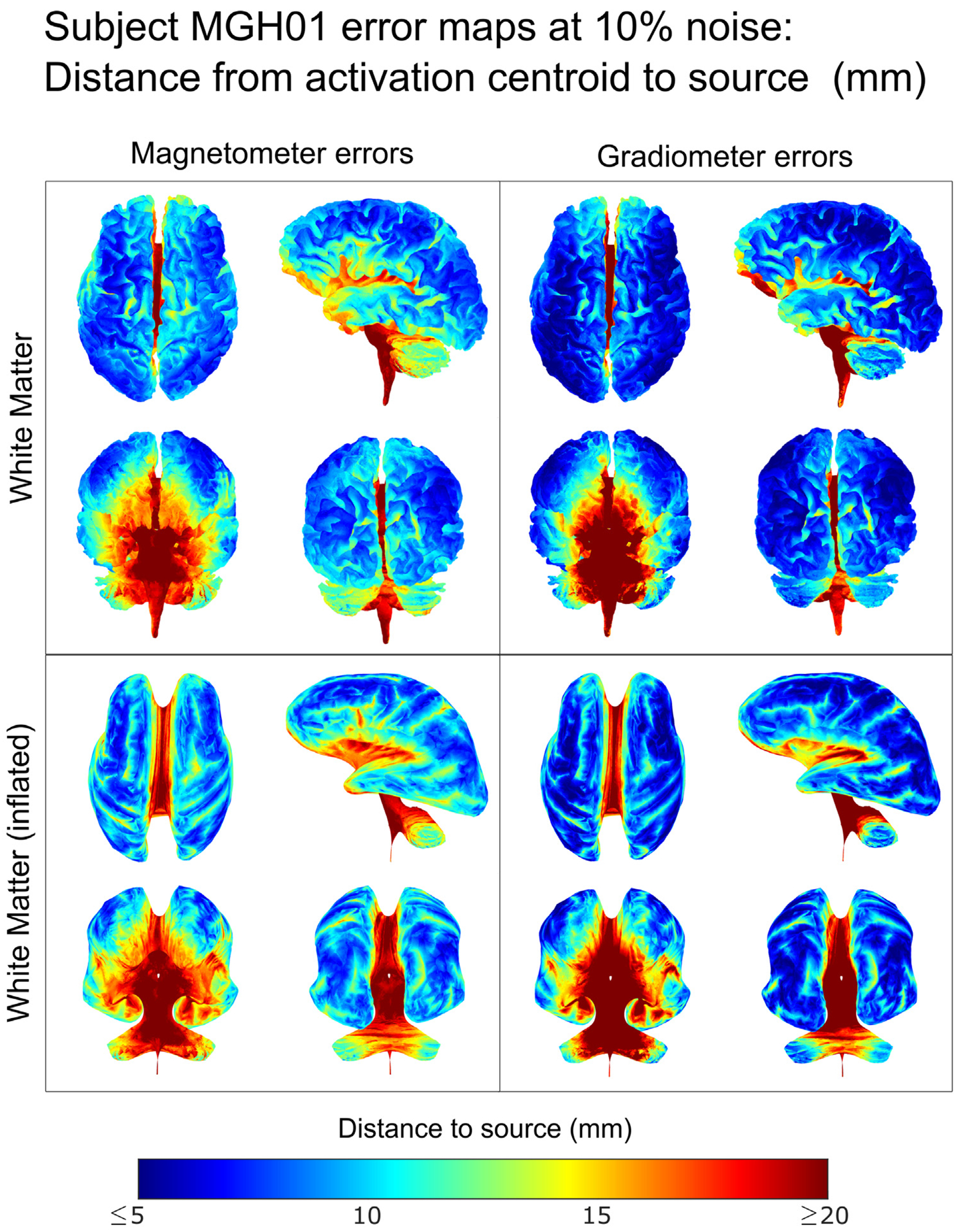
Error maps at 10% noise level (mm) for subject MGH01 on the white matter and inflated white matter surfaces. At each surface point, the average distance from the source to the centroid of estimated source strengths is interpolated from the exact computation of 2000 dipole locations chosen uniformly at random with 500 samples of noise at 10% level for each dipole location. In each box, we display different views of each tissue: top left — dorsal transverse view; top right — left lateral sagittal view; bottom left — anterior coronal view; bottom right — posterior coronal view.

**Fig. 10.**
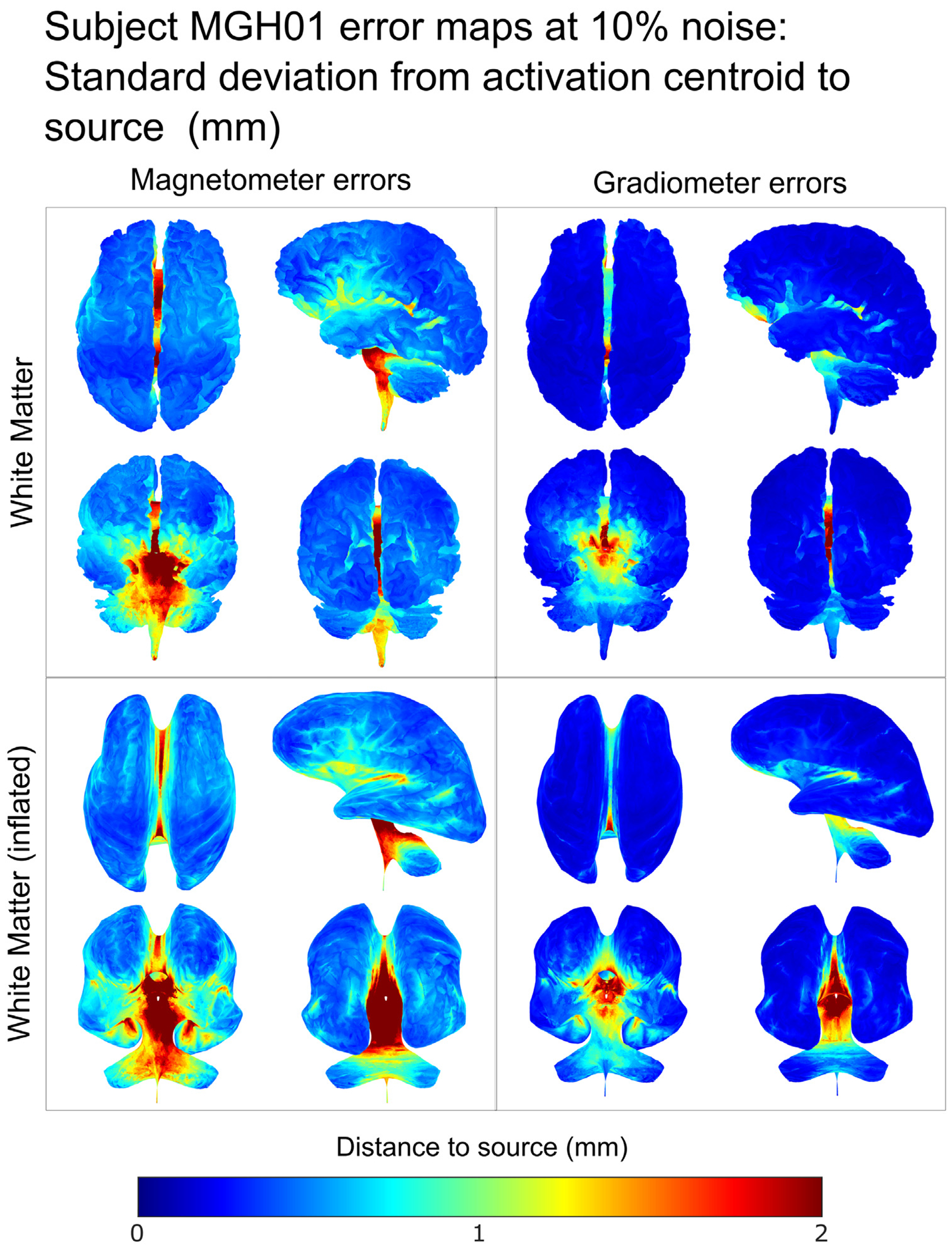
Error maps at 10% noise level (mm) for subject MGH01 on the white matter and inflated white matter surfaces. At each surface point, the standard deviation of distance from the source to the centroid of estimated source strengths is interpolated from the exact computation of 2000 dipole locations chosen uniformly at random with 500 samples of noise at 10% level for each dipole location. In each box, we display different views of each tissue: top left — dorsal transverse view; top right — left lateral sagittal view; bottom left — anterior coronal view; bottom right — posterior coronal view.

Figs. 9 and 10 show the source localization error maps for subject MGH01, with Fig. 9 showing the average source localization error on this subjects cortex and inflated white matter, generated from 2000 dipole sources across the cortex and interpolating the results. Fig. 10 shows the corresponding standard deviations. Additional error maps for the remaining subjects can be found in Supplement C.

## 4. Discussion

### Modeling aspects.

Even though we made use of ~250,000 sources (WM triangles) in each of our BEM-FMM inverse models, we mention that it is possible to refine and smooth the WM mesh to obtain even denser source spaces. Our decision for the number of sources/triangles we used is motivated by (1) the fact that this is the standard number given by headreco; and (2) computational constraints imposed by the more extensive direct computations of synthetic data.

We carried preliminary tests with reciprocal solutions using WM meshes of up to 1 million triangles, as well as more recent head models including a higher number of tissue layers (IT’IS Foundation, 2025a). The computation times for the reciprocal BEM-FMM do not increase prohibitively: on a typical academic computer cluster node (32 GB RAM and 12 cores, AMD architecture), each basis function with 250,000 sources took an average of 2.5 min to compute. The corresponding averages for 1,000,000 and 4,000,000 sources were 8 min and 45 min, respectively. This corresponds to an average computation time of 6 × 10^−4^, 4.8 × 10^−4^, and 6.7 × 10^−4^ seconds per source in each case, suggesting an approximately linear scaling. By parallelization of the basis function computations across different nodes, we were able to compute the forward model with 4,000,000 sources in under 1.5 h. We mention that we made no attempts to optimize our forward computation pipeline. Improving the implementation, one could significantly decrease these average computation times, although the time scaling behavior would likely remain the same. A boxplot figure showing additional details on these computation times can be found in Supplement C. Further analyses using more detailed models and additional experimental data are interesting directions for future research.

### Analysis of experimental data.

From Figs. 5 and 6, we see that the reciprocal BEM-FMM source reconstruction at the M20 peak yields activation regions similar to those of the 3-layer MNE-Python. Additionally, we find that activation centroids and peaks are located in similar regions for both reconstruction methods across all subjects. We find that regions of higher activation thresholds appear deep within the Brodmann Area 3b for both source reconstruction methods, consistent with the regions reported in previous experiments (Kawamura et al., 1996; Kakigi et al., 2000).

Because our inverse models makes use of a denser source space, it does not require smoothing and these higher-definition source estimates provide sharper contrasts following sulcal wall patterns. In contrast, the coarser source models of MNE-Python require smoothing, since without it the reconstructed source strength density would appear as a sparse cloud of points, hindering the interpretability of the results. This smoothing process has the effect of blurring areas with isolated peaks. This effect may or may not be desirable, depending on the experiment.

The AUC values in Table 3 indicate good inverse model performance for all methods considered, with our reciprocal BEM-FMM inverse model being slightly favored on average; mostly due to a few cases where other methods fail to give a good solution. We consider that the reason why we observe only a marginal improvement is that the somatosensory fields constitute a particularly favorable case for source reconstruction and, as such, simplified models will give a good performance. This being said, we note that the results in Table 3 need to be interpreted with care, as we only have an estimated ground truth and we transformed the source estimation problem into a binary classification problem. Additionally, any formal comparison between other models and our reciprocal BEM-FMM inverse models would require numerical experiments using the same source models and meshes, in such a way that the model discrepancies on source estimation of synthetic data can only be attributed to the use of different forward models. Likewise, the effect of increasing the number of sources (or equivalently, the spatial density of the source space within the cortex) needs to be tested using identical head models. Our results in this paper are meant to be interpreted as a validation for our proposed method. The study of the accuracy of inverse models as a function of source space density remains as an interesting avenue for future research.

### Model performance of MNE inverse models derived from reciprocal BEM-FMM for auditory evoked fields.

If instead we considered more challenging evoked fields, such as auditory evoked fields (AEFs), we would observe further improvements over low resolution models. In our companion study (Drumm et al., 2025), we applied the methodology developed in this paper to localize binaural AEFs. We observed that the inverse minimum norm estimation models derived from high-resolution reciprocal BEM-FMM provided estimates of much higher focality and accuracy than those of MNE-Python using low-resolution 1- or 3-layer direct BEM. Possible explanations for this behavior are (1) the primary components of AEFs are generated in the Heschl gyri, which are located in the temporal lobes where low-resolution models fail to accurately describe the participant’s anatomy; and (2) binaural AEFs are generated on the Heschl gyri of both hemispheres, and a higher-density source space may be able to better distinguish between two distant simultaneous sources.

### Evaluation against synthetic data.

The results in Table 4 indicate that our inverse method is robust against the addition of increasing levels of noise. Comparing Tables 4a and 4b, one can see that the distance errors associated to 75% threshold activation centroids are, on average, smaller than those associated to source estimation peaks. This effect is also observed in the error maps. It appears that to localize an isolated source, the centroid of the reconstructed region activation may be a more accurate estimate of the true source location than the peak.

The distance error maps in Fig. 9 and Supplement C confirm that source reconstruction of sources in the deepest parts of the cortex can be better recovered from magnetometer data than from gradiometer data. We also observe that sources along the central sulcus are among those with the lowest average distance errors. The standard deviation of the error maps in Fig. 10 and in Supplement C show that magnetometer source reconstructions have a higher degree of uncertainty than those using gradiometer signals. These errormaps also reveal a higher level of uncertainty in the deeper cortical regions.

## 5. Conclusions

We proposed, implemented, and tested an accelerated reciprocal forward solver for MEG source localization based on BEM-FMM. This solver can be used to create gain matrices involving millions of sources without sacrificing the resolution of the entire computational mesh. This opens the possibility to employ high-density source spaces in MEG source estimation. Inverse models derived using reciprocal BEM-FMM, along with high-resolution tissue meshes and source spaces, yielded highly focal activation estimates on Brodmann area 3b for SEFs from 5 healthy participants. These results align with previous literature on localization of SEFs and validate the applicability of our forward modeling approach to source estimation.

Evaluation against synthetic data from dipoles placed on the primary somatosensory cortex shows that our high-resolution inverse models perform well even with the addition of noise to the source signal. The average distance error was 6 mm, with a standard deviation ranging from 0.03 to 0.69 mm across different signal-to-noise ratio (SNR) levels.

## Supplementary Material

1

2

3

## Figures and Tables

**Table 1:** Parameters selected for the TMS forward computations. The symbol $\mu_{A}$ denotes the average triangle area in the mesh. The absence of a unit label in the “Value” column indicates a dimensionless parameter.

Parameter	Value
FMM precision for ρ	1 × 10^−4^
FMM precision for $E^{i}$	1 × 10^−4^
GMRES relative residual tolerance	1 × 10^−4^
Neighbor radius for local integral correction	$2\sqrt{\mu_{A}}$ [m]
Characteristic of the coil pulse waveform: $\partial i_{j}/\partial t$	1 [A s^−1^]

**Table 2:** Conductivities and number of triangles in each tissue layer for the reciprocal BEM-FMM and MNE-Python models.

(a) Tissue layers in the reciprocal BEM-FMM inverse model, their associated conductivities, their average number of triangles, and the bibliographic references for these values.^†^ The conductivity value of the ventricles is chosen to coincide with that of the CSF.			
Tissue	Conductivity [S m^−1^]	avg. tri.	Reference

Skin	0.25	231,000	Truong et al. (2012)
Skull	0.01	237,000	Wagner et al. (2004)
CSF	1.654	126,000	Wagner et al. (2004)
GM	0.275	347,000	Wagner et al. (2004), Thielscher and Wichmann (2009)
WM	0.126	384,000	Wagner et al. (2004), Thielscher and Wichmann (2009)
Ventricles	1.654	14,800	Wagner et al. (2004) ^†^
Eyes	0.5	5070	Gabriel et al. (1996), Opitz et al. (2015)


(b) Tissue layers in the 3-layer BEM MNE-Python and 3-layer isotropic FEM inverse models and their conductivities. In the case of the 1-layer BEM, we used only the inner-skull surface with conductivity value 0.33. Each of the BEM layers contains 20,480 triangular faces, and the FEM models have an average of 250,000 elements.		
Tissue name	Conductivity [S m^−1^]	Reference
Skin	0.25	Truong et al. (2012)
Outer skull	0.01	Wagner et al. (2004)
Inner skull	0.33	Vorwerk et al. (2019)

**Table 3:** The AUC values of the source localization results for every subject model using Reciprocal BEM-FMM inverse models, MNE-Python (1 and 3 layer), and 3 layer isotropic FEM inverse models from Brainstorm. AUC values closer to 1 indicate better model performance.

(a) AUC values for source localization using only the magnetometer recordings at the M20 peak.						
Magnetometer sensors	MGH01	MGH02	MGH03	MGH04	MGH05	Mean

R-BEM-FMM	0.8972	0.8113	0.8414	0.9365	0.9265	0.8826
MNE-Python (1 layer)	0.8991	0.7862	0.7823	0.8056	0.8350	0.8216
MNE-Python (3 layer)	0.8863	0.7692	0.7802	0.8026	0.8326	0.8142
FEM	0.9053	0.7885	0.8551	0.8485	0.8407	0.8476


(b) AUC values for source localization using only the gradiometer recordings at the M20 peak.						
Gradiometer sensors	MGH01	MGH02	MGH03	MGH04	MGH05	Mean

R-BEM-FMM	0.9033	0.7614	0.8250	0.9556	0.9374	0.8765
MNE-Python (1 layer)	0.8726	0.8245	0.8070	0.7489	0.8507	0.8207
MNE-Python (3 layer)	0.8723	0.7906	0.7973	0.7694	0.8581	0.8175
FEM	0.8524	0.7443	0.8198	0.8862	0.8409	0.8287

**Table 4:** Aggregated distance error statistics for source estimation of simulated data (at various noise levels) for a dipole placed in the primary somatosensory cortex: The distance from centroids of the estimated region of activation (thresholded at 75%), and from the peak of activation, to the true source location are computed and averaged for each subject.

(a) Centroid-to-source distance error statistics for source localization based on simulated magnetometer and gradiometer signals.						
SNR	Magnetometer data			Gradiometer data		

	Mean	Median	Std	Mean	Median	Std

Noiseless	6.67	–	–	6.09	–	–
81	6.49	6.52	0.05	6.71	6.67	0.07
27	6.50	6.46	0.11	6.74	6.67	0.13
9	6.51	6.42	0.18	6.77	6.71	0.22
3	6.76	6.51	0.75	6.71	6.79	0.58
2	7.17	6.78	1.19	6.80	6.83	0.85
1.5	7.90	6.20	3.40	7.08	5.94	5.58


(b) Peak-to-source distance error statistics for source localization based on simulated magnetometer and gradiometer signals.						
SNR	Magnetometer data			Gradiometer data		

	Mean	Median	Std	Mean	Median	Std

Noiseless	10.08	–	–	9.92	–	–
81	10.38	10.38	0.00	9.54	9.75	0.22
27	10.38	10.38	0.03	9.53	9.31	0.24
9	10.44	10.38	0.30	9.58	9.45	0.37
3	10.72	10.38	1.63	9.64	9.54	0.47
2	11.32	10.44	2.55	9.68	9.48	0.80
1.5	12.31	10.56	6.14	11.63	9.64	10.17

## Data Availability

Data will be made available on request.

## Appendix A. Supplementary data

Supplementary material related to this article can be found online at https://doi.org/10.1016/j.neuroimage.2025.121452.
