# Supplementary material for: High-definition MEG source estimation using the reciprocal boundary element fast multipole method
Source: Neuroimage. Author manuscript; Available in PMC 2025 Nov 12. (PMC12606592; doi:10.1016/j.neuroimage.2025.121452)
Supplement: 1 [file NIHMS2116705-supplement-1.pdf]

Supplement A. MEG-TMS Reciprocity Theorem in Frequency Domain and in Time Domain

A MEG-TMS reciprocity theorem in frequency domain and in time domain

A.1 Lorentz reciprocity theorem

The initial reciprocity relation, or Lorentz reciprocity theorem, in integral form without magnetic sources (cf. Equation (A.1) in Heller and van Hulsteyn 1992),

$$\int_V \mathbf{J}_1 \cdot \mathbf{E}_2 d\mathbf{r} = \int_V \mathbf{J}_2 \cdot \mathbf{E}_1 d\mathbf{r}, \tag{A1}$$

implicitly uses the symmetry of Maxwell's equations. It is derived from the full set of Maxwell equations, written exclusively in phasor form and without any additional assumptions. By peforming a set of multiplications and subtractions (Plonsey 1972; Balanis 2012), Equation A1 relates vector phasors of two arbitrary sinusoidal impressed current source distributions, $\mathbf{J}_1$ and $\mathbf{J}_2$, operating at the same frequency ω to the vector phasors of their respective electric fields, $\mathbf{E}_1$ and $\mathbf{E}_2$. The integration is done over the entire space where the current source distributions exist. According to Lorrain and Corson 1988, the Lorentz reciprocity theorem (e.g. its simplified form in a source-free region) "is paradoxical because it establishes a relation between two unrelated electromagnetic fields".

A.2 Singular impressed harmonic currents in cosine form

In the present application, $\mathbf{J}_1(\mathbf{r})$ is the impressed phasor point current source located at $\mathbf{r}_1$ within the cortex with vector dipole moment $\mathbf{p}_1$ $[\mathrm{A\,m}]$. On the other hand, $\mathbf{J}_2(\mathbf{r})$ is the impressed phasor line current of an MEG sensor (a gradiometer or magnetometer) outside the head operating as a TMS (transcranial magnetic stimulation) coil. Without loss of generality, both independent impressed current sources are assumed to be in phase. This makes it possible to set their phasors as two real numbers simultaneously. The corresponding real-valued impressed current densities can then be written in the form (cf. Equation (A.2) in Heller and van Hulsteyn 1992)

$$\begin{aligned} \mathbf{J}_1(\mathbf{r}, t) &= \mathbf{J}_1(\mathbf{r}) \cos \omega t = \mathbf{p}_1 \delta(\mathbf{r} - \mathbf{r}_1) \cos \omega t, \\ \mathbf{J}_2(\mathbf{r}, t) &= \mathbf{J}_2(\mathbf{r}) \cos \omega t = I_0 \mathbf{t}_2(\ell) \delta(\mathbf{r} - \mathbf{r}_2(\ell)) \cos \omega t, \end{aligned} \tag{A2}$$

with all constants being real. Here, $\mathbf{r}_2(\ell)$ is a vector running along the contour C of the coil conductor (i.e. the MEG sensor) (where $\ell \in C$), $\mathbf{t}_2(\ell)$ is unit tangent to the coil conductor, I_0 is the coil current amplitude $[\mathrm{A}]$, and δ is the delta function.

A.3 Transformation of Lorentz reciprocity equation by expressing $\mathbf{E}_1(\mathbf{r})$

After substitution of Equation A2 into Equation A1, we obtain (cf. Equation (A.4) in (Heller and van Hulsteyn 1992))

$$\mathbf{p}_1 \cdot \mathbf{E}_2(\mathbf{r}_1) = I_0 \int_C \mathbf{t}_2(\ell) \cdot \mathbf{E}_1(\mathbf{r}) d\ell = I_0 \int_C d\ell \cdot \mathbf{E}_1(\mathbf{r}). \tag{A3}$$

In the quasi-static case, the MEG cortical dipole creates an in-phase conduction current and the associated electric field distribution within the head with the same $\cos \omega t$ dependence, similar to DC steady state. Simultaneously, a time-varying magnetic flux everywhere in space will be generated by this total current distribution in the conducting head. To the highest order of magnitude, the magnetic flux is in phase with the current, again similar to DC steady state. Therefore, we can set for the real-valued magnetic flux $\mathbf{B}_1(\mathbf{r}, t)$,

$$\mathbf{B}_1(\mathbf{r}, t) = \mathbf{B}_1(\mathbf{r}) \cos \omega t, \tag{A4}$$

where $\mathbf{B}_1(\mathbf{r})$ is a purely-real vector phasor. A nonzero circulation of phasor electric field $\mathbf{E}_1$ along the closed contour L, or a nontrivial line integral on the right-hand side of Equation A3 is due to Faraday's laws of induction, which reads in terms of the phasors,

$$\int_C d\ell \cdot \mathbf{E}_1 = -i\omega \int_S \mathbf{n} \cdot \mathbf{B}_1(\mathbf{s}) d\mathbf{s}. \tag{A5}$$

Substitution of Equation A5 into Equation A3 yields (cf. Equations (A.5) and (A.6) of Heller and van Hulsteyn 1992),

$$\mathbf{p}_1 \cdot \mathbf{E}_2(\mathbf{r}_1) = -I_0(i\omega) \int_S \mathbf{n} \cdot \mathbf{B}_1(\mathbf{s}) d\mathbf{s}. \tag{A6}$$

A.4 Transformation of Equation A6 by expressing $\mathbf{E}_2(\mathbf{r})$

In order to satisfy Equation A6 with the real phasor $\mathbf{B}_1(\mathbf{r})$ and the real constants $\mathbf{p}_1$ and I_0, respectively, the phasor $\mathbf{E}_2(\mathbf{r}_1)$ of the TMS electric field must be purely imaginary. Let us show that this is really the case.

The TMS coil electric field $\mathbf{E}_2(\mathbf{r})$ on the left-hand side of Equation A1 and A3 is due to the coil generator current $\mathbf{J}_2(\mathbf{r})$ in Equation A2. This field has two components: a solenoidal part and a conservative part. In terms of phasor magnetic vector potential $\mathbf{A}_2(\mathbf{r})$ and phasor electric scalar potential $\varphi_2(\mathbf{r})$, one obtains (cf. p. 261 in Balanis 2012),

$$\mathbf{E}_2(\mathbf{r}) = -i\omega \mathbf{A}_2(\mathbf{r}) - \nabla \varphi_2(\mathbf{r}). \tag{A7}$$

In the quasi-static approximation, the magnetic vector potential is expressed by a static formula through the TMS coil current and without the effect of the conducting head. It is strictly in phase with the coil generator current in Equation A2, so its phasor $\mathbf{A}_2(\mathbf{r})$ is a real function,

$$\mathbf{A}_2(\mathbf{r}) = \frac{\mu_0}{4\pi} I_0 \int_C \frac{d\ell}{|\mathbf{r} - \mathbf{r}_2(\ell)|}. \tag{A8}$$

The phasor electric scalar potential $\varphi_2(\mathbf{r})$ is that of electric charges induced at conductivity boundaries in response to the primary solenoidal coil field component, $-i\omega\mathbf{A}_2(\mathbf{r})$. It must therefore be of the same form, $-\nabla\varphi_2(\mathbf{r}) = -i\omega\nabla\Phi_2(\mathbf{r})$, with $\Phi_2(\mathbf{r})$ being real. From Equation A7, one then has,

$$\mathbf{E}_2(\mathbf{r}) = -i\omega\mathbf{E}_2^r(\mathbf{r}), \;\; \mathbf{E}_2^r(\mathbf{r}) = \mathbf{A}_2(\mathbf{r}) + \nabla\Phi_2(\mathbf{r}), \tag{A9}$$

with the real phasor $\mathbf{E}_2^r(\mathbf{r})$. Substituting Equation A9 into Equation A6, one arrives at the final result

$$\mathbf{p}_1 \cdot \mathbf{E}_2^r(\mathbf{r}_1) = I_0 \int_S \mathbf{n} \cdot \mathbf{B}_1(\mathbf{s}) d\mathbf{s} \tag{A10}$$

A.5 Reciprocity theorem in time domain for harmonic excitation

Multiplying both sides of Equation A10 by $\cos\omega t$, one can obtain the corresponding time-domain result for harmonic excitation,

$$\mathbf{p}_1 \cdot \mathbf{E}_2(\mathbf{r}_1, t) = I(t) \int_S \mathbf{n} \cdot \mathbf{B}_1(\mathbf{s}) d\mathbf{s}, \tag{A11}$$

where

- $I(t) = I_0 \cos\omega t$ is a harmonic TMS coil current; and
- $\mathbf{E}_2^r(\mathbf{r}, t) = \mathbf{E}_2^r(\mathbf{r}) \cos\omega t$ is a harmonic time integral, $\mathbf{E}_2(\mathbf{r}, t) = -\frac{\partial}{\partial t}\mathbf{E}_2^r(\mathbf{r}, t)$, of the TMS coil field $\mathbf{E}_2(\mathbf{r}, t)$.

Differentiating Equation A11 over time leads to,

$$\mathbf{p}_1 \cdot \mathbf{E}_2(\mathbf{r}, t) = -\frac{\partial I(t)}{\partial t} \int_S \mathbf{n} \cdot \mathbf{B}_1(\mathbf{r}) ds. \tag{A12}$$

This is a useful result stated previously in (Nummenmaa et al. 2013). It follows in particular that $\frac{\partial I}{\partial t} = -\omega I_0 \sin\omega t$ and $\mathbf{E}_2(\mathbf{r}, t) = \omega\mathbf{E}_2^r(\mathbf{r}) \sin\omega t$. Plugging these into Equation A12 gives us again Equation A11.

References

Balanis, Constantine A. (2012). *Advanced engineering electromagnetics*. 2nd ed. John Wiley & Sons.

Heller, L and D B van Hulsteyn (July 1992). "Brain stimulation using electromagnetic sources: theoretical aspects". In: *Biophys. J.* 63.1, pp. 129–138. DOI: 10.1016/S0006-3495(92)81587-4.

Lorrain, Paul and Dale R. Corson (1988). *Electromagnetic Fields and Waves*. 3rd ed. W. H. Freeman, pp. 502–503.

Nummenmaa, Aapo et al. (2013). "Comparison of spherical and realistically shaped boundary element head models for transcranial magnetic stimulation navigation". In: *Clin. Neurophysiol.* 124.10, pp. 1995–2007. DOI: https://doi.org/10.1016/j.clinph.2013.04.019.

Plonsey, R (May 1972). "Capability and limitations of electrocardiography and magnetocardiography". en. In: *IEEE Trans. Biomed. Eng.* 19.3, pp. 239–244.
