# Supplementary material for: High-definition MEG source estimation using the reciprocal boundary element fast multipole method
Source: Neuroimage. Author manuscript; Available in PMC 2025 Nov 12. (PMC12606592; doi:10.1016/j.neuroimage.2025.121452)
Supplement: 2 [file NIHMS2116705-supplement-2.pdf]

Supplement B. Derivation of the Inverse Operator

A Derivation of the inverse operator

Here, we give a derivation of the inverse operator M of Equation **??** based on error minimization; this is adapted (correcting a minor logical flaw) from the derivation of (Liu 2000).

Let $\mathbf{b}$ be the vector of measured MEG signals, $\mathbf{x}$ the vector of dipole strengths, and $\mathbf{n}$ a noise vector. Then, the measured MEG signals are related to the dipole strengths $\mathbf{x}$ by the following equation

$$\mathbf{b} = L\mathbf{x} + \mathbf{n}. \tag{B1}$$

Suppose that the dipole strengths follow a multivariate Gaussian distribution of zero mean and covariance matrix R (the source-covariance matrix). Likewise, assume that the noise follows a multivariate Gaussian with zero-mean and covariance matrix $\lambda^2\Sigma$ (the noise-covariance matrix, with a regularization parameter). Then, by definition,

$$\mathbf{x}\mathbf{x}^\intercal = R, \text{ and } \mathbf{n}\mathbf{n}^\intercal = \lambda^2\Sigma. \tag{B2}$$

We calculate the linear operator M that minimizes the expected error:

$$\mathrm{Err}_M = \|M\mathbf{b} - \mathbf{x}\|^2. \tag{B3}$$

Substitute the expression for $\mathbf{b}$ of Equation B2 into the expected error to find

$$\begin{aligned}
\mathrm{Err}_M &= \|M(L\mathbf{x} + \mathbf{n}) - \mathbf{x}\|^2 \\
&= \|(ML - I)\mathbf{x} + M\mathbf{n}\|^2 \\
&\leq \|(ML - I)\mathbf{x}\|^2 + \|M\mathbf{n}\|^2 \\
&= \mathrm{tr}((ML - I)\mathbf{x}\mathbf{x}^\intercal(ML - I)^\intercal) + \mathrm{tr}(M\mathbf{n}\mathbf{n}^\intercal M^\intercal) \\
&= \mathrm{tr}((ML - I)R(ML - I)^\intercal) + \mathrm{tr}(\lambda^2 M\Sigma M^\intercal),
\end{aligned} \tag{B4}$$

where tr is the trace operator, i.e. the sum of the diagonal elements of the matrix. In particular, we have that the trace is linear and for a matrix A, $\mathrm{tr}(A) = \mathrm{tr}(A^\intercal)$. Expanding the term $((ML - I)R(ML - I))^\intercal$, we find

$$\begin{aligned}
\mathrm{tr}((ML - I)R(ML - I)^\intercal) &= \mathrm{tr}(MLRL^\intercal M^\intercal - MLR - RL^\intercal M^\intercal + R) \\
&= \mathrm{tr}(MLRL^\intercal M^\intercal - 2RL^\intercal M^\intercal + R).
\end{aligned} \tag{B5}$$

Here, we used the fact that $\mathrm{tr}(MLR) = \mathrm{tr}((MLR)^\intercal) = \mathrm{tr}(RL^\intercal M^\intercal)$. So we have

$$\mathrm{Err}_W \leq \mathrm{tr}(MLRL^\intercal M^\intercal - 2RL^\intercal M^\intercal + R) + \mathrm{tr}\,\lambda^2 M\Sigma M^\intercal. \tag{B6}$$

To find the operator M that minimizes the error, we take the gradient with respect to M in the right-hand-side of the equation above and equate to 0, obtaining:

$$0 = 2MLRL^\intercal - 2RL^\intercal + 2\lambda^2 M\Sigma. \tag{B7}$$

From here, we obtain

$$M(LRL^\intercal + \lambda^2\Sigma) = RL^\intercal. \tag{B8}$$

Hence,

$$M = RL^\intercal(LRL^\intercal + \lambda^2\Sigma)^{-1}. \tag{B9}$$

References

Liu, Arthur K. (2000). “Spatiotemporal brain imaging”. PhD thesis. Massachusetts Institute of Technology. URL: https://dspace.mit.edu/handle/1721.1/8963.
