# Supplementary material for: High-definition MEG source estimation using the reciprocal boundary element fast multipole method
Source: Neuroimage. Author manuscript; Available in PMC 2025 Nov 12. (PMC12606592; doi:10.1016/j.neuroimage.2025.121452)
Supplement: 3 [file NIHMS2116705-supplement-3.pdf]

Supplement C. Additional Tables and Figures

A Additional Tables and Figures

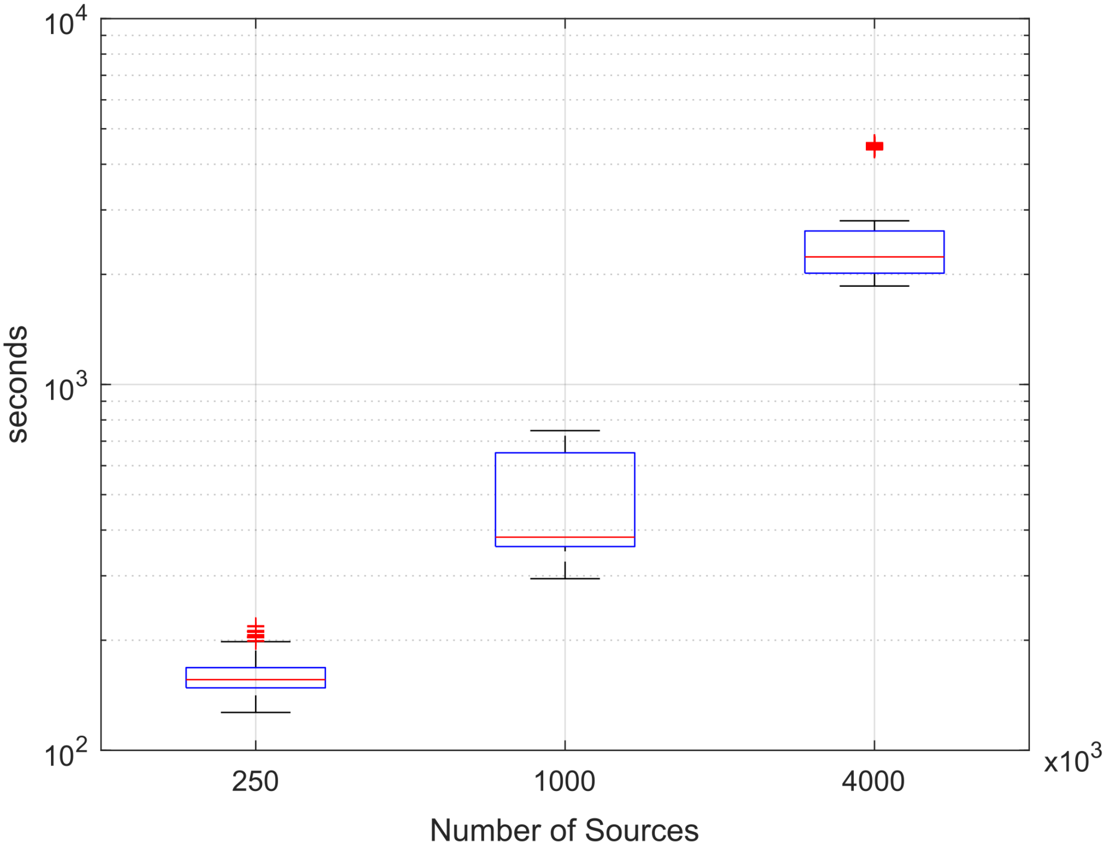

Figure 1: Box plots denoting time taken to compute the basis functions (rows of the lead field matrix) for subject MGH01 with 250,000, 1,000,000, and 4,000,000 sources, respectively. Computations were performed on Turing cluster nodes with 32GB RAM, 12 cores, and AMD architecture. Red lines within the boxes denote the mean computation time, and red lines outside the boxes denote outliers.

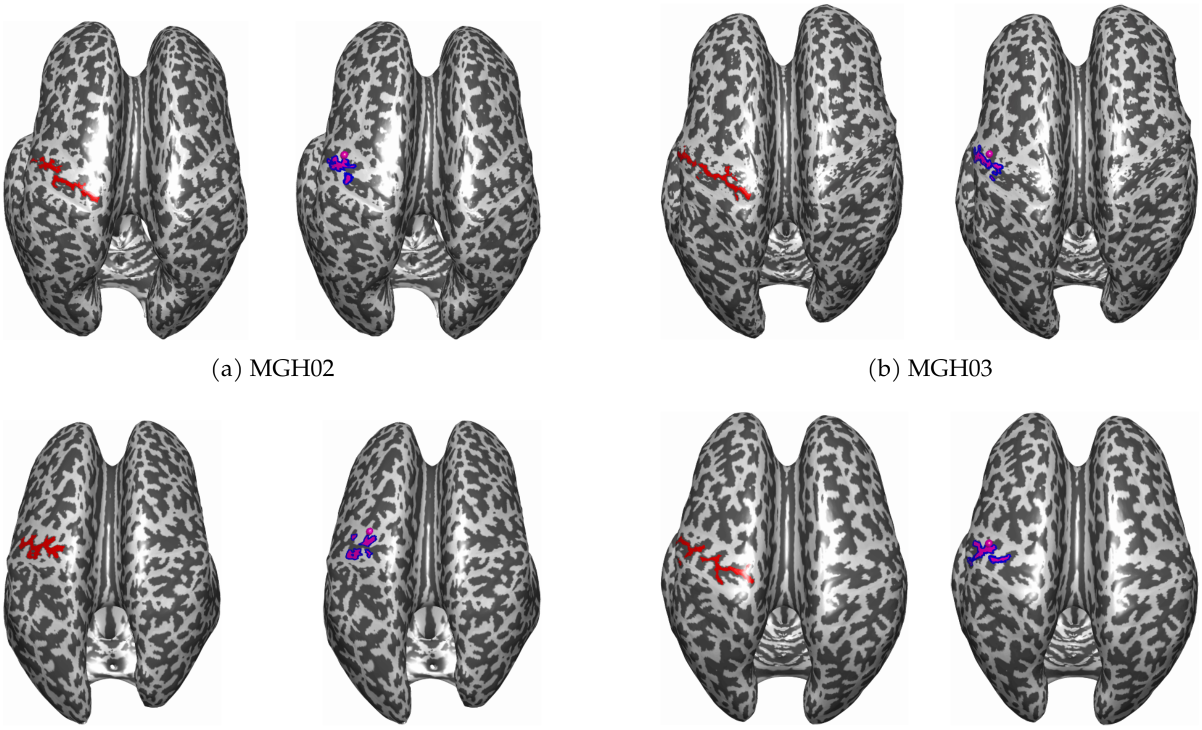

(a) MGH02 (b) MGH03

(c) MGH04 (d) MGH05

Figure 2: (left) The points on the inflated white matter surface of each subject identifying the central sulcus are shown in red. (right) The tagged point denoting the M1 hand is denoted by the magenta sphere. The target region, shown in magenta, is deterimined by locating the points in the central sulcus nearest to the tagged M1 hand so that 40% of the central sulcus points are covered. The blue region denotes the 2mm distance demarcating the classifier boundary.

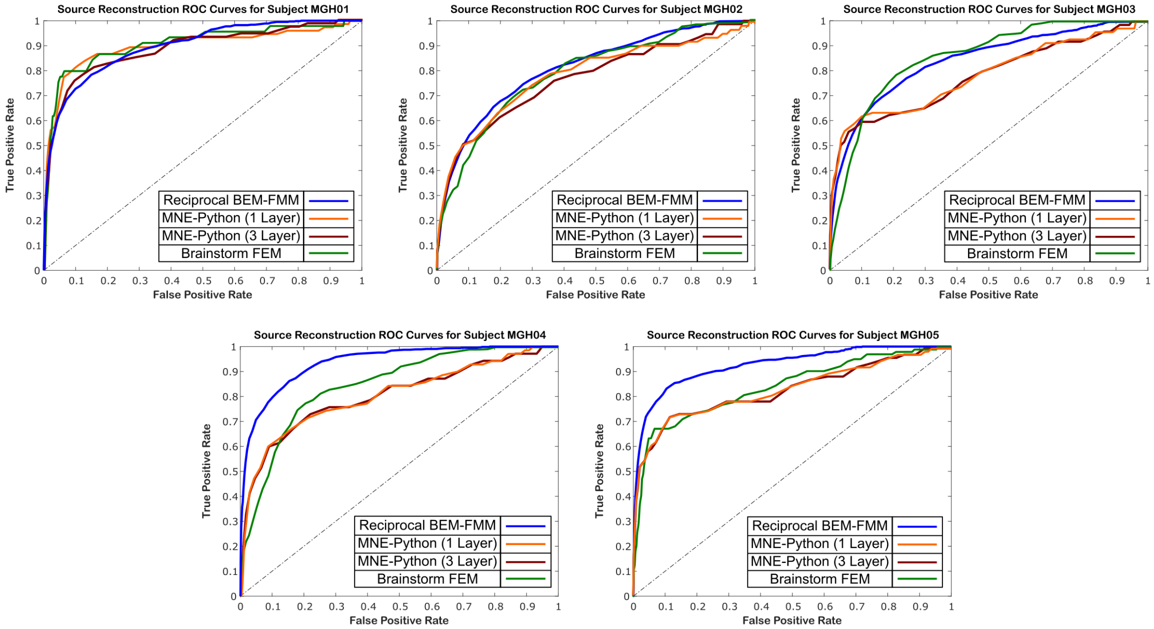

(a) Models which consider only the magnetometer sensor signals.

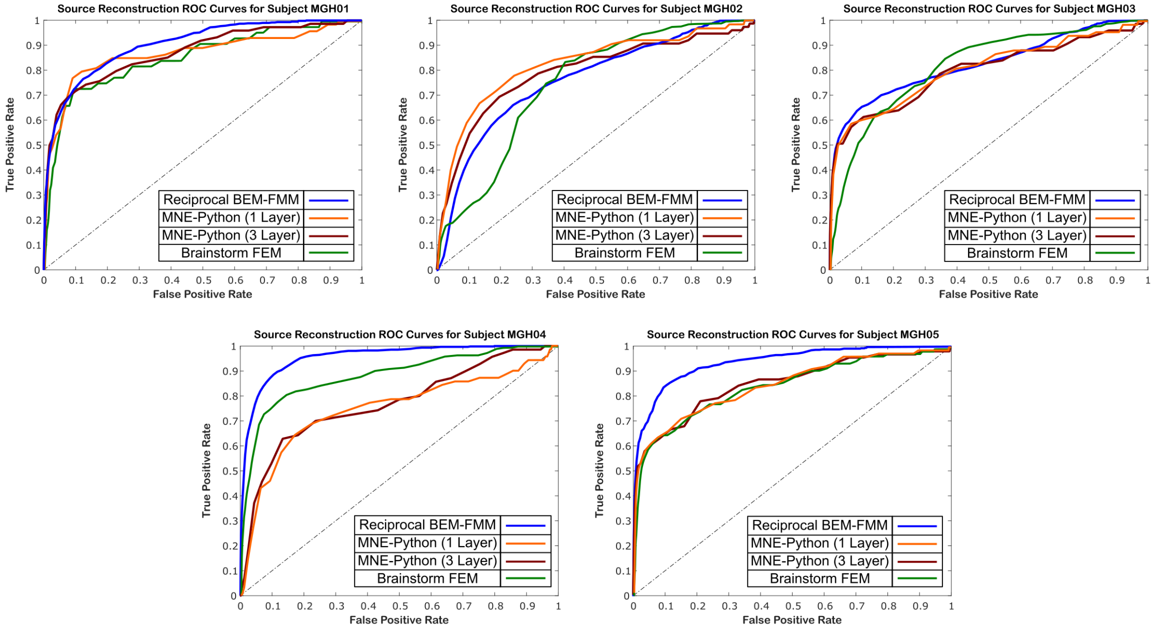

(b) Models which consider only the gradiometer signals.

Figure 3: The ROC curves for the source reconstruction results of every subject using Reciprocal BEM-FMM, 1 and 3 layer BEM (MNE-Python), and FEM (Brainstorm). The black dashed line indicates the "random chance" model.

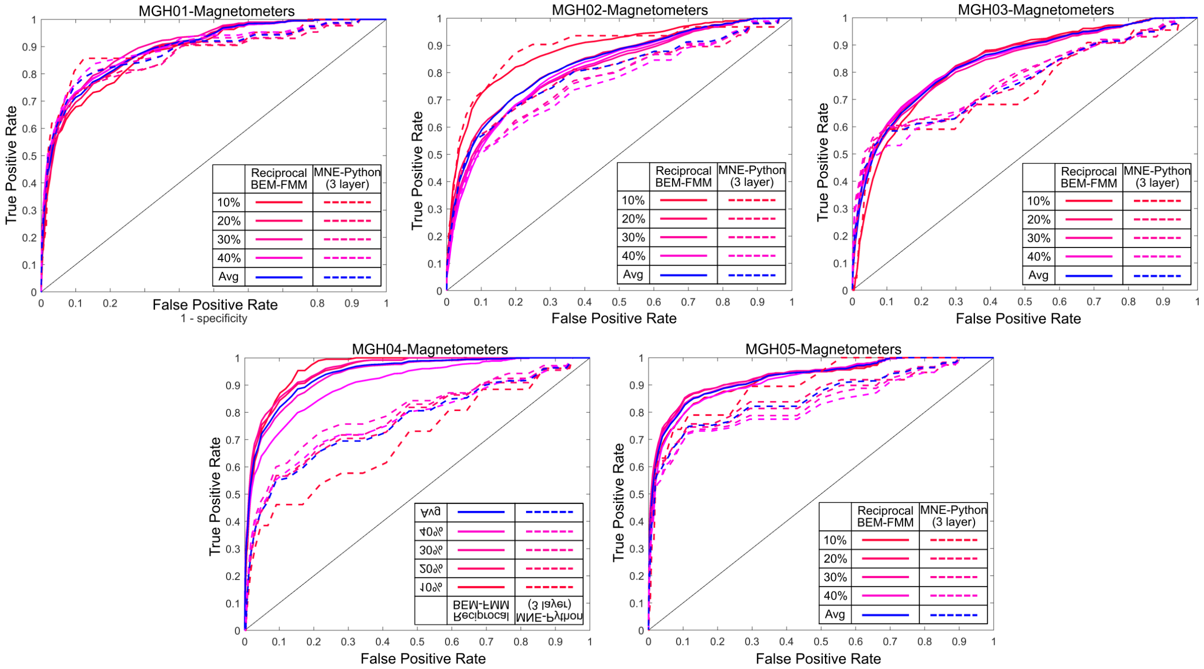

(a) Models which consider only the magnetometer sensor signals.

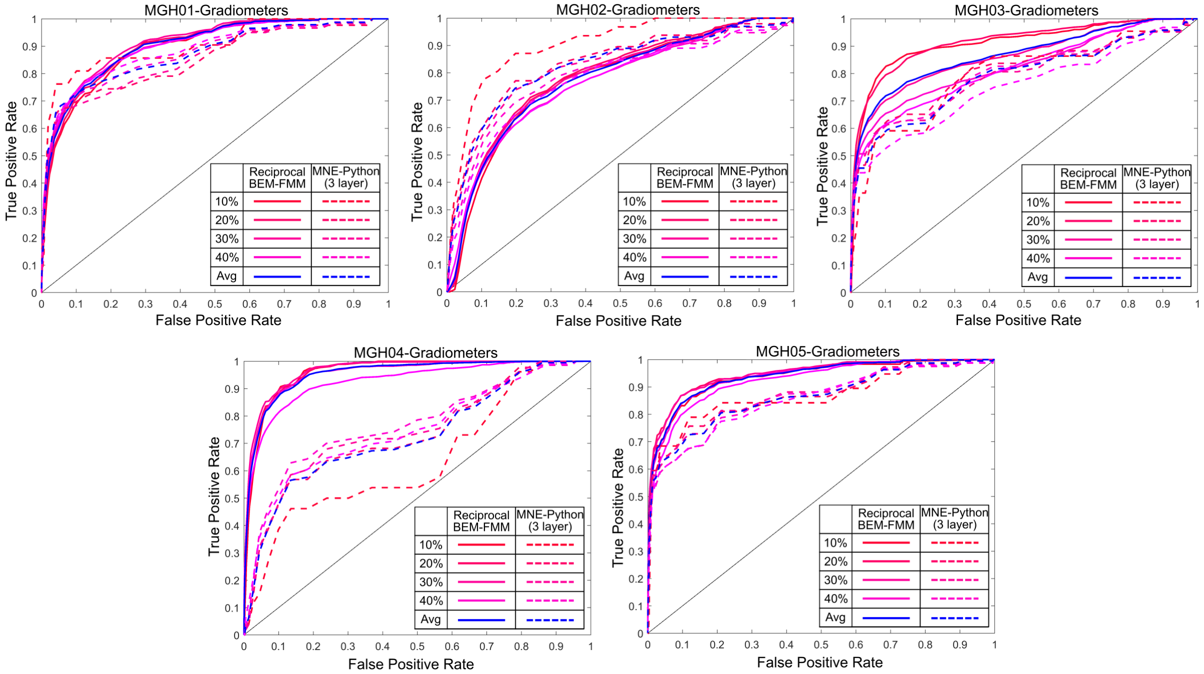

(b) Models which consider only the gradiometer signals.

Figure 4: The ROC curves for the source reconstruction results of every subject using Reciprocal BEM-FMM and MNE-Python (3 layer) across varying percentages of central sulcus area covered nearest to the M1-hand.

Table 1: Statistical results for centroid-to-source distance error with noiseless and noisy simulated data for every MGH subject. Centroids are taken from the set of sources with strengths within 75-100% of the maximum strength.

**MGH01**						
	Magnetometer Data			Gradiometer Data		
SNR	Mean	Median	Std	Mean	Median	Std
Noiseless	4.28	-	-	4.34	-	-
81	2.93	3.07	0.15	6.03	5.97	0.07
27	2.88	2.77	0.14	6.03	5.97	0.07
9	2.91	2.77	0.18	6.03	5.97	0.15
3	3.73	3.07	1.17	5.50	5.97	1.10
2	4.32	3.71	1.40	5.45	5.97	1.17
1.5	5.62	4.91	6.04	8.33	6.00	10.38

**MGH02**						
	Magnetometer Data			Gradiometer Data		
SNR	Mean	Median	Std	Mean	Median	Std
Noiseless	12.50	-	-	11.01	-	-
81	13.12	13.12	0.03	11.27	11.16	0.22
27	13.06	13.12	0.10	11.34	11.16	0.31
9	12.93	12.94	0.28	11.32	11.16	0.42
3	12.75	12.86	0.61	11.25	11.16	0.73
2	12.94	12.88	1.21	11.30	11.20	1.11
1.5	13.47	12.94	2.61	11.85	11.63	2.99

**MGH03**						
	Magnetometer Data			Gradiometer Data		
SNR	Mean	Median	Std	Mean	Median	Std
Noiseless	6.09	-	-	5.89	-	-
81	5.69	5.68	0.04	5.32	5.32	0.00
27	5.74	5.68	0.15	5.32	5.32	0.01
9	5.82	5.68	0.21	5.29	5.32	0.15
3	5.87	5.76	0.24	5.28	5.32	0.27
2	5.90	5.98	0.41	5.41	5.32	0.44
1.5	6.17	5.98	4.10	6.85	5.68	6.26

**MGH04**						
	Magnetometer Data			Gradiometer Data		
SNR	Mean	Median	Std	Mean	Median	Std
Noiseless	5.00	-	-	3.97	-	-
81	4.89	4.88	0.04	4.79	4.75	0.07
27	4.97	4.88	0.14	4.82	4.75	0.09
9	5.03	4.88	0.16	4.85	4.93	0.10
3	5.38	4.93	1.35	4.95	4.93	0.18
2	6.37	5.19	2.33	5.02	4.93	0.62
1.5	7.49	5.75	2.84	6.92	4.98	6.83

**MGH05**						
	Magnetometer Data			Gradiometer Data		
SNR	Mean	Median	Std	Mean	Median	Std
Noiseless	5.50	-	-	5.25	-	-
81	5.83	5.83	0.00	6.17	6.17	0.00
27	5.83	5.83	0.00	6.22	6.17	0.16
9	5.85	5.83	0.10	6.36	6.17	0.30
3	6.07	5.94	0.38	6.57	6.55	0.64
2	6.31	6.15	0.62	6.80	6.72	0.93
1.5	6.74	1.44	1.44	1.44	1.44	1.44

Table 2: Statistical results for peak-to-source distance error with noiseless and noisy simulated data for every MGH subject.

**MGH01**						
	Magnetometer Data			Gradiometer Data		
SNR	Mean	Median	Std	Mean	Median	Std
Noiseless	7.43	-	-	9.54	-	-
81	7.43	7.43	0.00	9.54	9.54	0.00
27	7.46	7.43	0.13	9.54	9.54	0.00
9	7.58	7.43	0.26	9.51	9.54	0.08
3	7.71	7.43	0.30	9.41	9.54	0.19
2	7.79	8.00	0.32	9.26	9.25	0.34
1.5	8.67	8.00	7.02	12.60	9.25	15.87

**MGH02**						
	Magnetometer Data			Gradiometer Data		
SNR	Mean	Median	Std	Mean	Median	Std
Noiseless	16.45	-	-	13.27	-	-
81	16.45	16.45	0.00	13.27	13.27	0.00
27	16.45	16.45	0.00	13.28	13.27	0.12
9	16.62	16.45	1.12	13.53	13.27	0.45
3	18.01	16.45	3.01	13.65	13.27	0.60
2	18.51	16.45	3.42	13.79	13.27	1.74
1.5	18.99	16.58	5.07	15.26	14.06	7.62

**MGH03**						
	Magnetometer Data			Gradiometer Data		
SNR	Mean	Median	Std	Mean	Median	Std
Noiseless	11.12	-	-	8.43	-	-
81	11.12	11.12	0.00	8.43	8.43	0.00
27	11.12	11.12	0.00	8.43	8.43	0.00
9	11.12	11.12	0.01	8.43	8.43	0.00
3	10.75	11.12	0.76	8.44	8.43	0.07
2	10.37	10.85	1.06	8.49	8.43	0.20
1.5	10.68	10.41	7.78	10.01	8.43	10.48

**MGH04**						
	Magnetometer Data			Gradiometer Data		
SNR	Mean	Median	Std	Mean	Median	Std
Noiseless	6.69	-	-	7.59	-	-
81	6.09	6.09	0.00	6.55	7.59	1.09
27	6.09	6.09	0.00	6.48	5.42	1.09
9	6.09	6.09	0.00	6.43	6.09	1.05
3	6.75	6.09	3.33	6.47	6.53	1.03
2	9.73	6.09	7.12	6.52	6.53	1.24
1.5	13.02	7.59	8.77	9.94	6.53	16.39

**MGH05**						
	Magnetometer Data			Gradiometer Data		
SNR	Mean	Median	Std	Mean	Median	Std
Noiseless	8.70	-	-	10.76	-	-
81	10.79	10.79	0.00	9.92	9.92	0.00
27	10.79	10.79	0.00	9.92	9.92	0.00
9	10.79	10.79	0.10	10.00	9.92	0.26
3	10.40	10.79	0.73	10.26	9.92	0.45
2	10.18	10.79	0.85	10.33	9.92	0.47
1.5	10.21	10.20	2.06	10.32	9.92	0.51

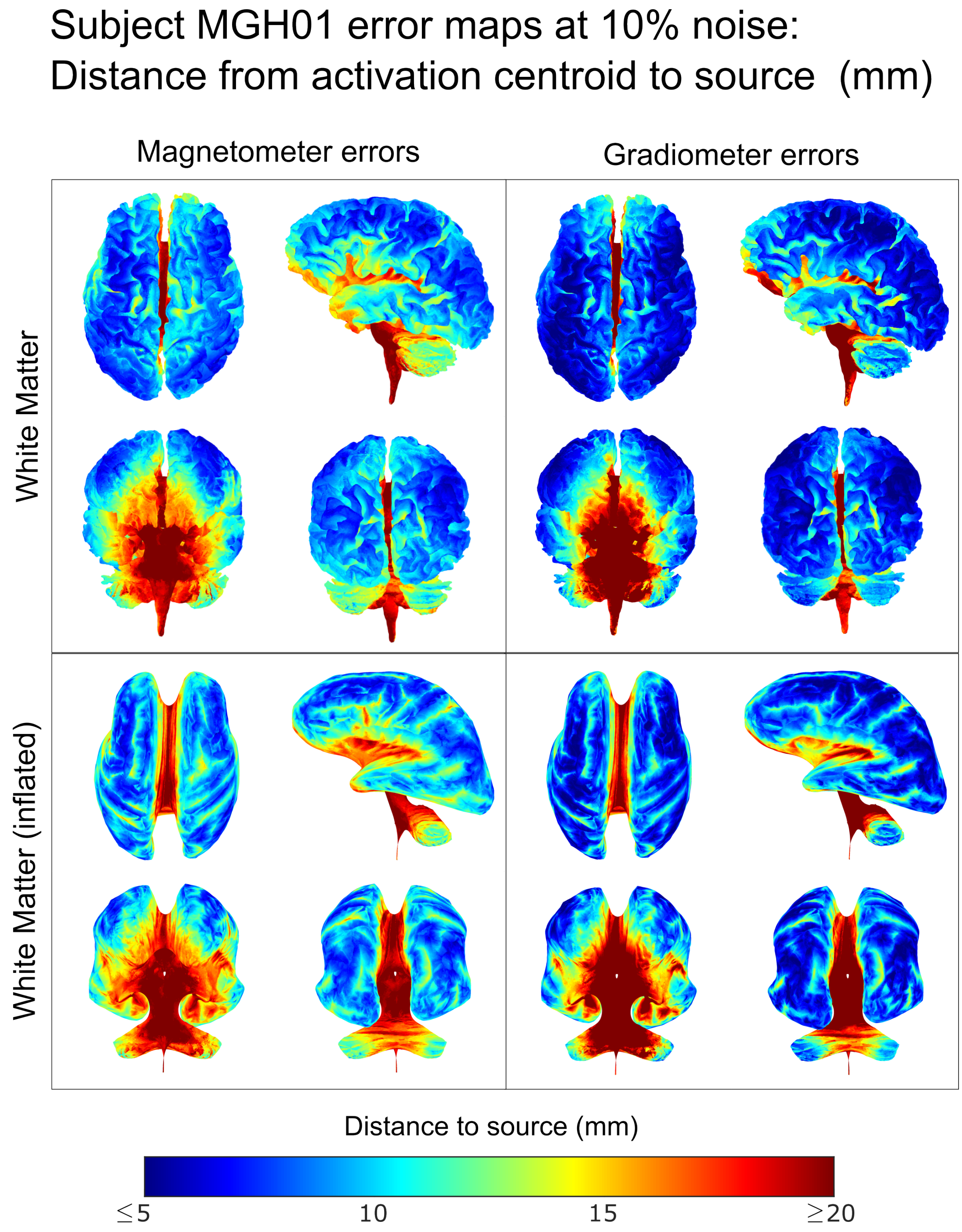

Figure 5: Error maps at 10% noise level (mm) for subject MGH01 on the white matter and inflated white matter surfaces.

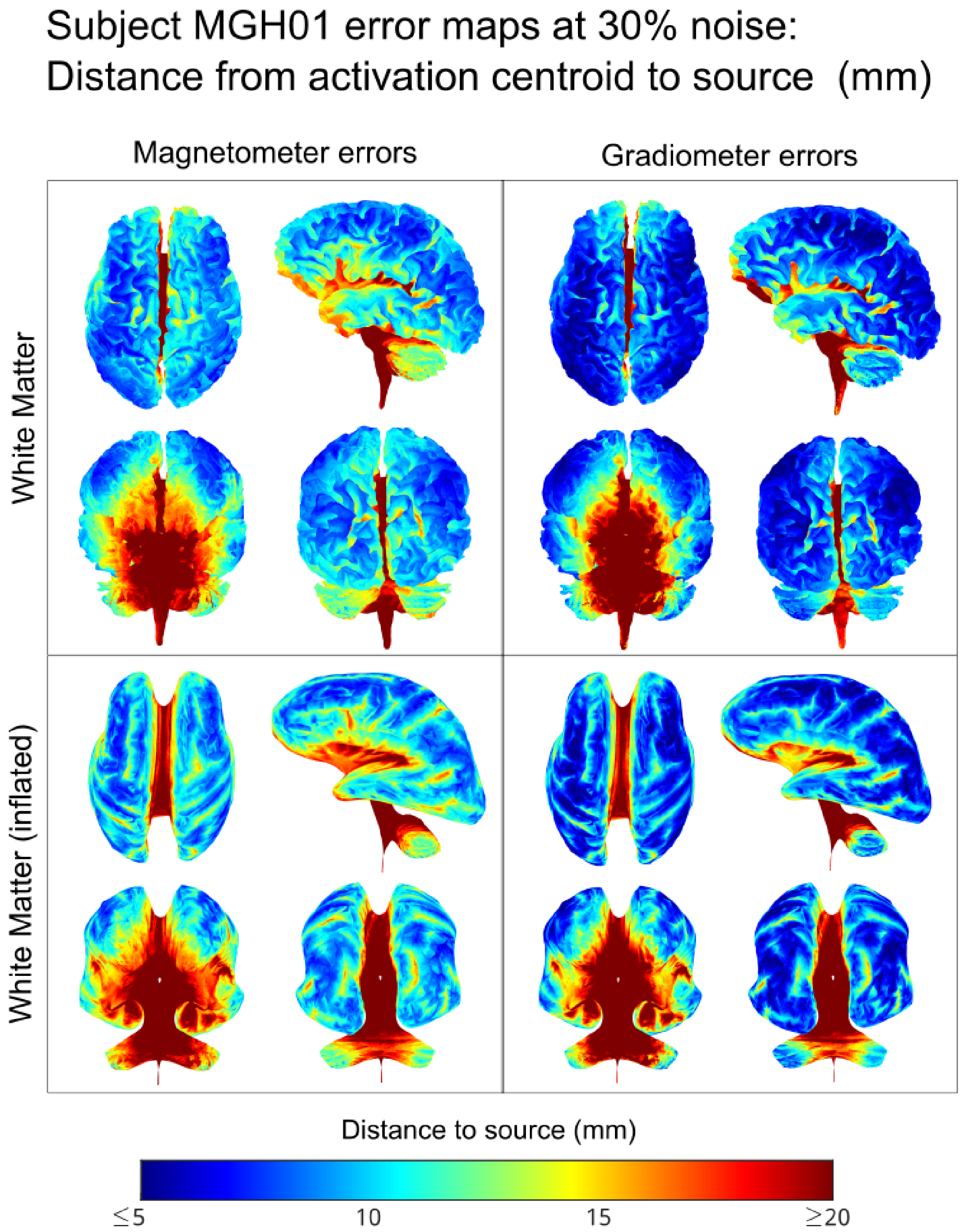

Figure 6: Error maps at 30% noise level (mm) for subject MGH01 on the white matter and inflated white matter surfaces.

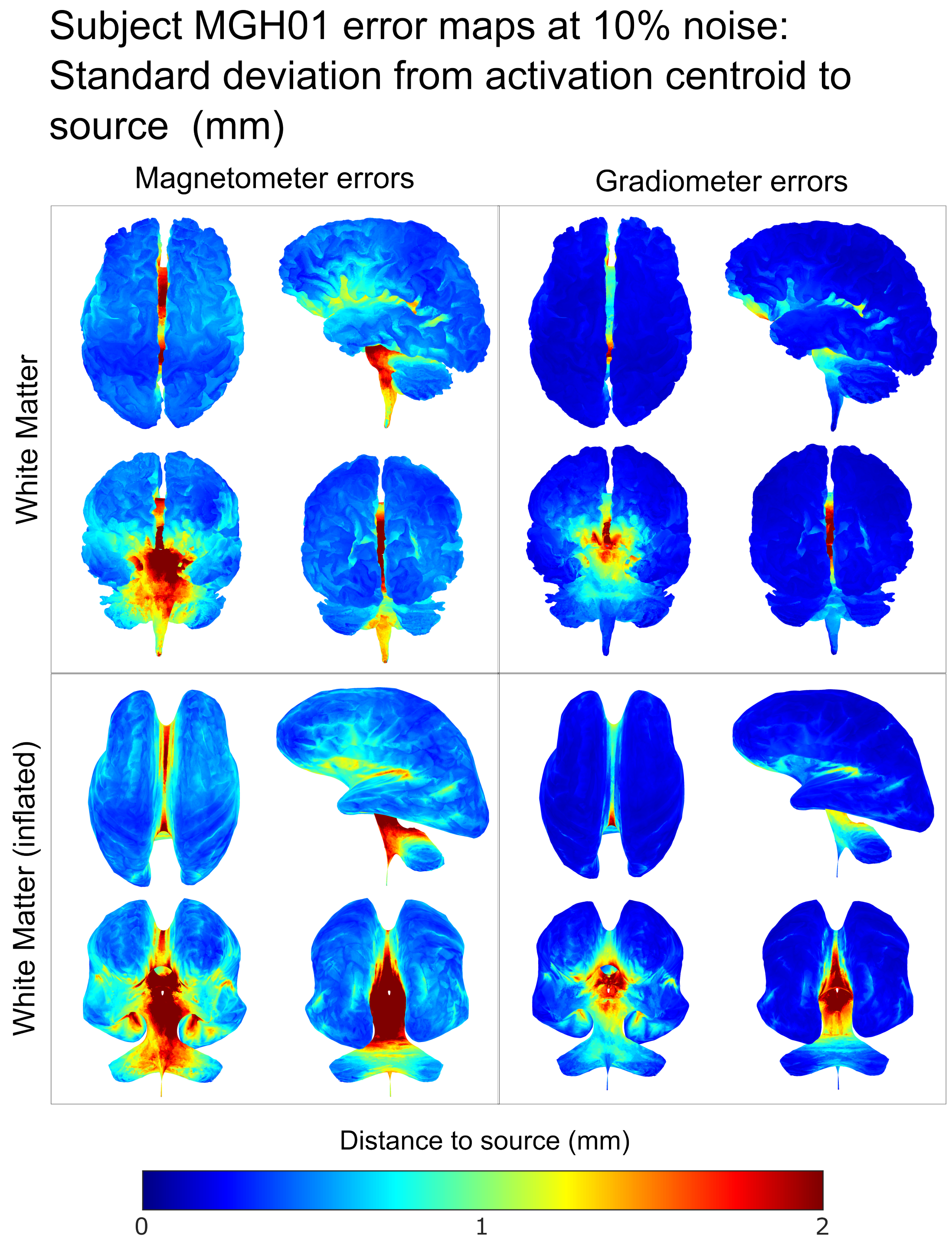

Figure 7: Error maps (standard deviation) at 10% noise level (mm) for subject MGH01 on the white matter and inflated white matter surfaces.

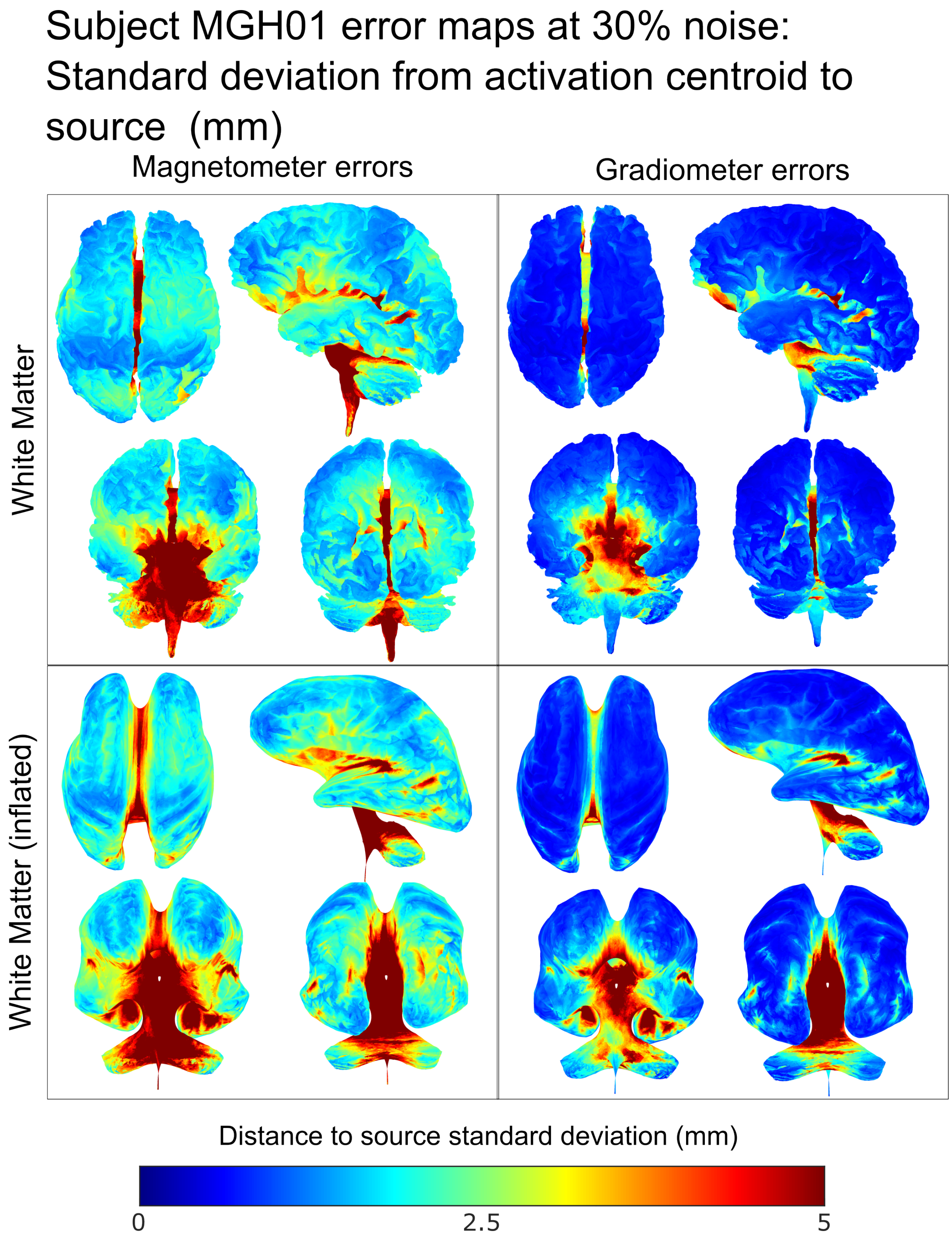

Figure 8: Error maps (standard deviation) at 30% noise level (mm) for subject MGH01 on the white matter and inflated white matter surfaces.

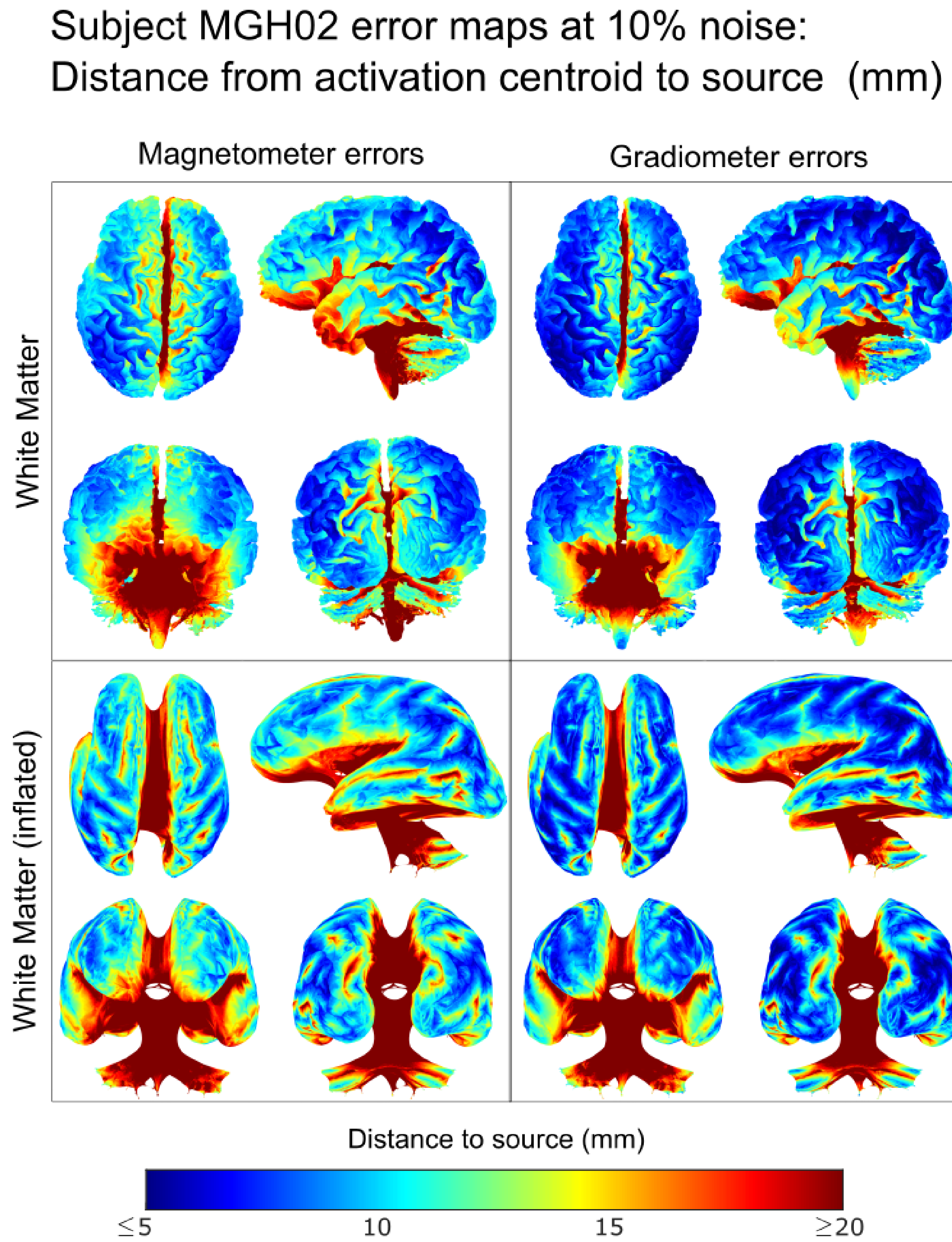

Figure 9: Error maps at 10% noise level (mm) for subject MGH02 on the white matter and inflated white matter surfaces.

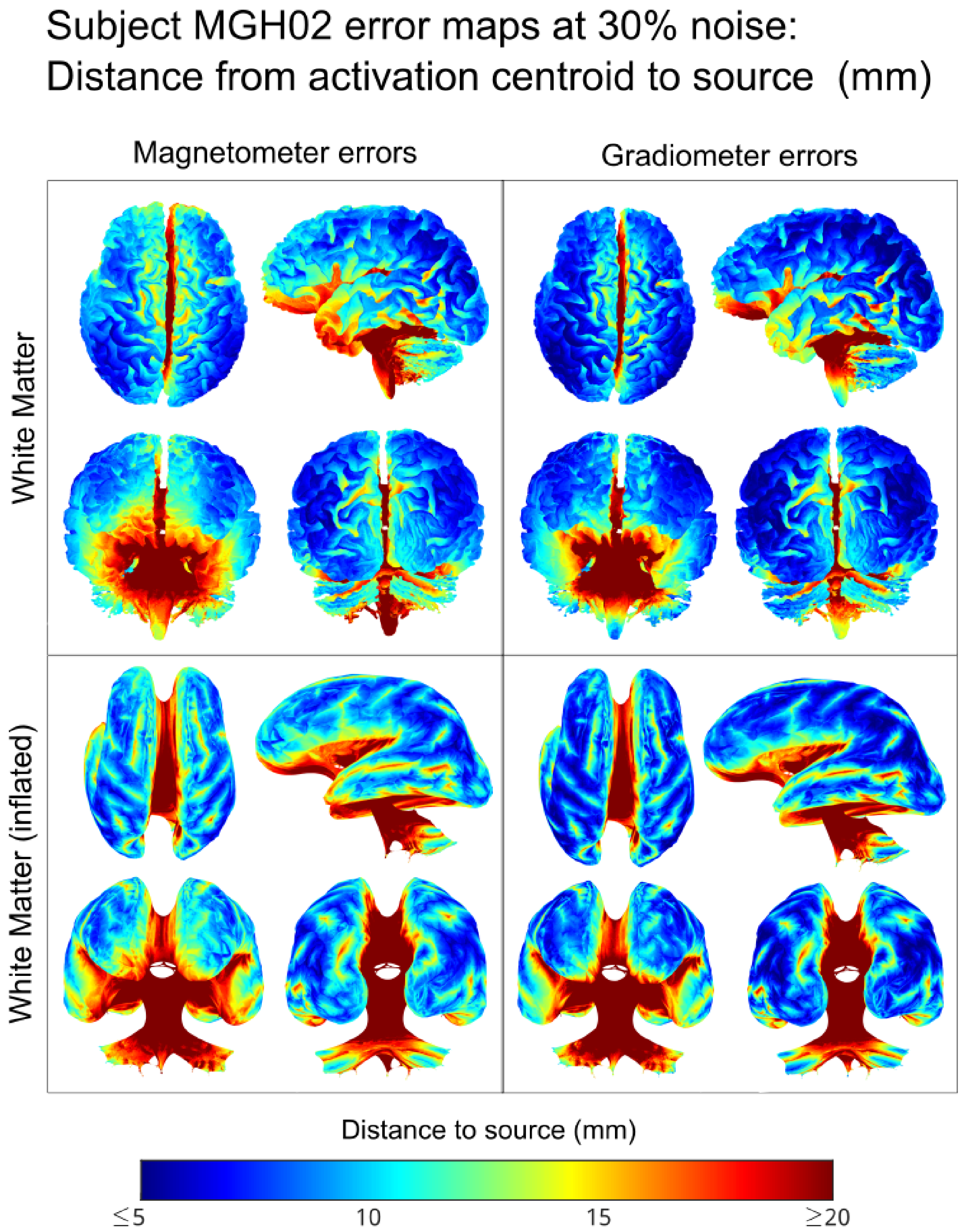

Figure 10: Error maps at 30% noise level (mm) for subject MGH02 on the white matter and inflated white matter surfaces.

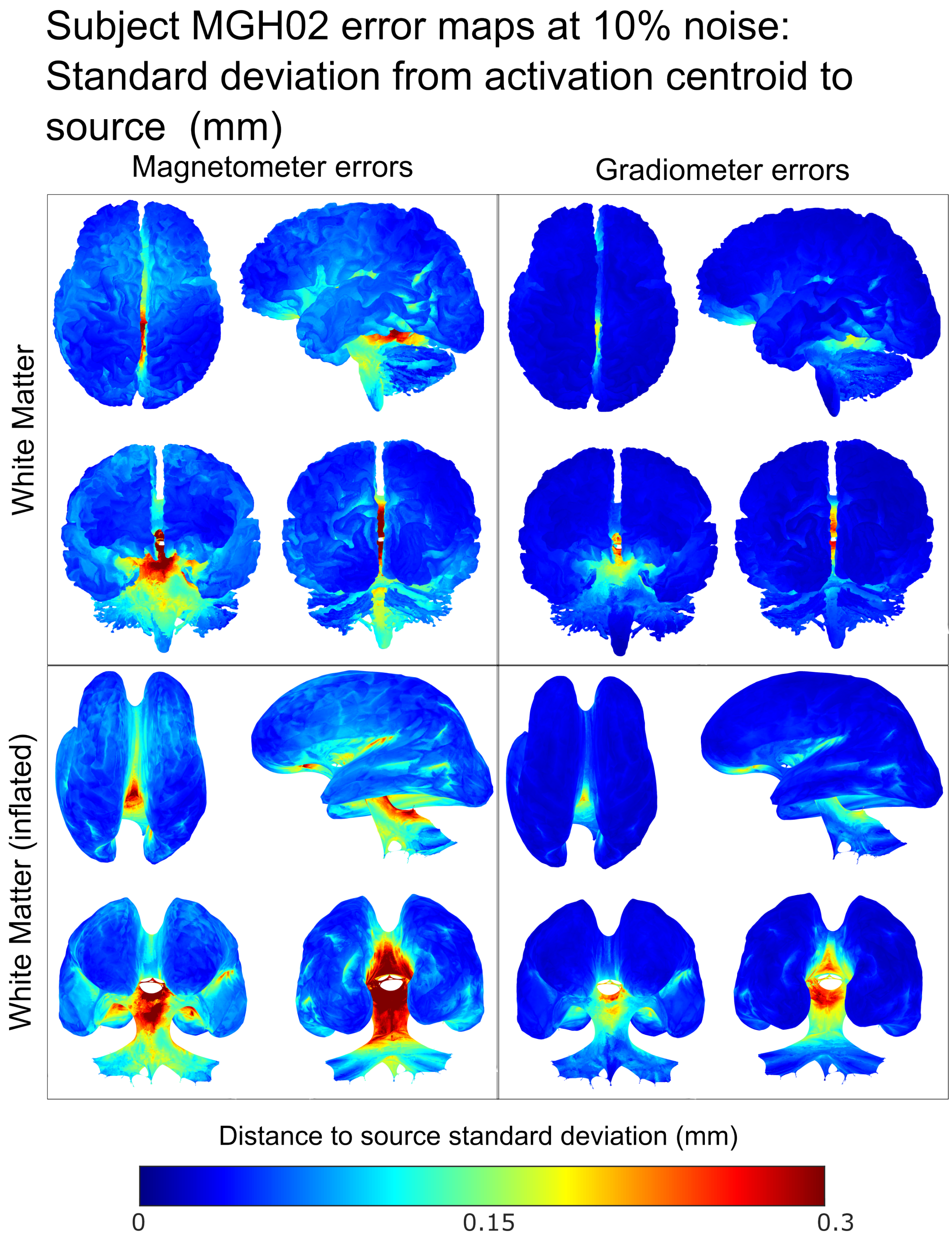

Figure 11: Error maps (standard deviation) at 10% noise level (mm) for subject MGH02 on the white matter and inflated white matter surfaces.

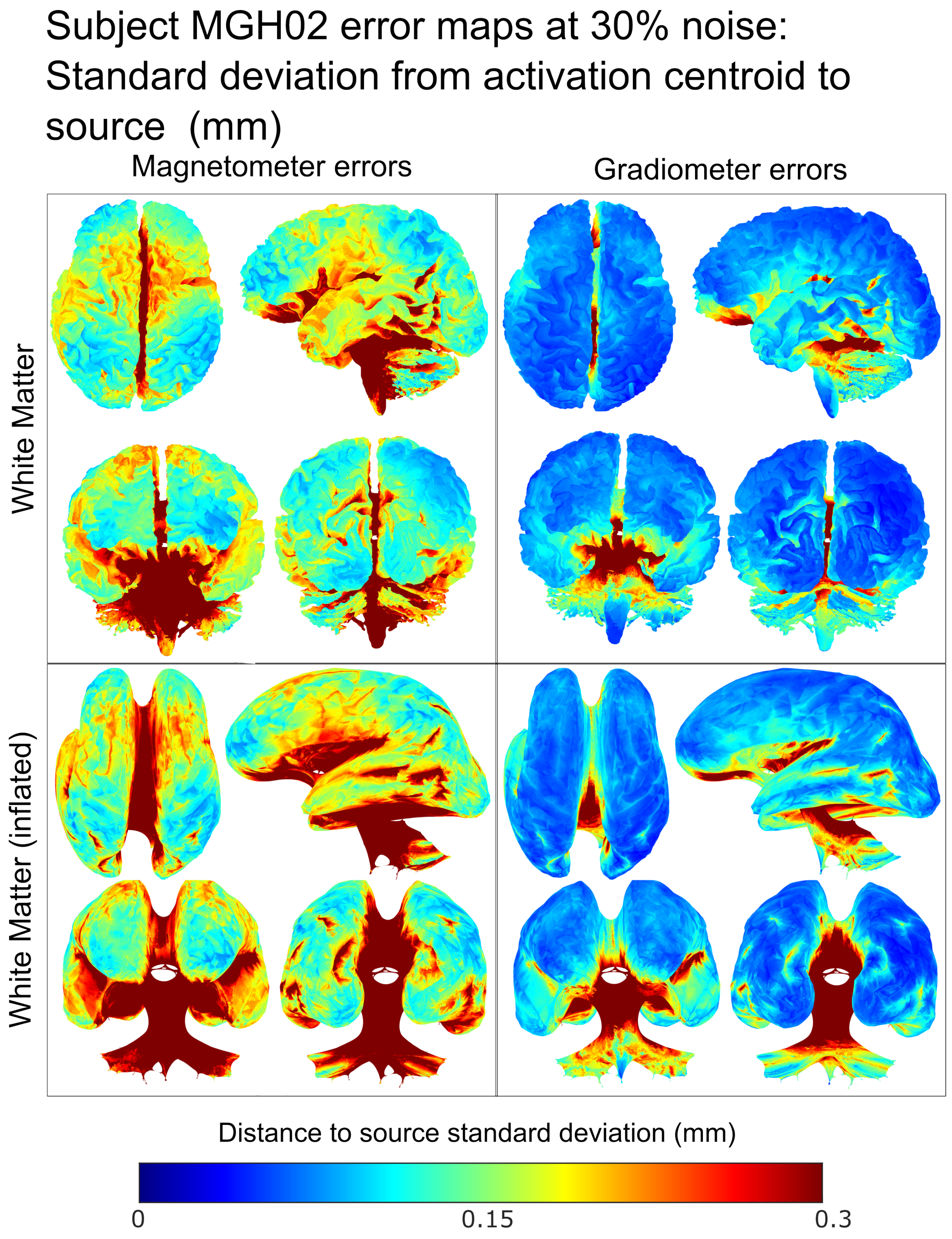

Figure 12: Error maps (standard deviation) at 30% noise level (mm) for subject MGH02 on the white matter and inflated white matter surfaces.

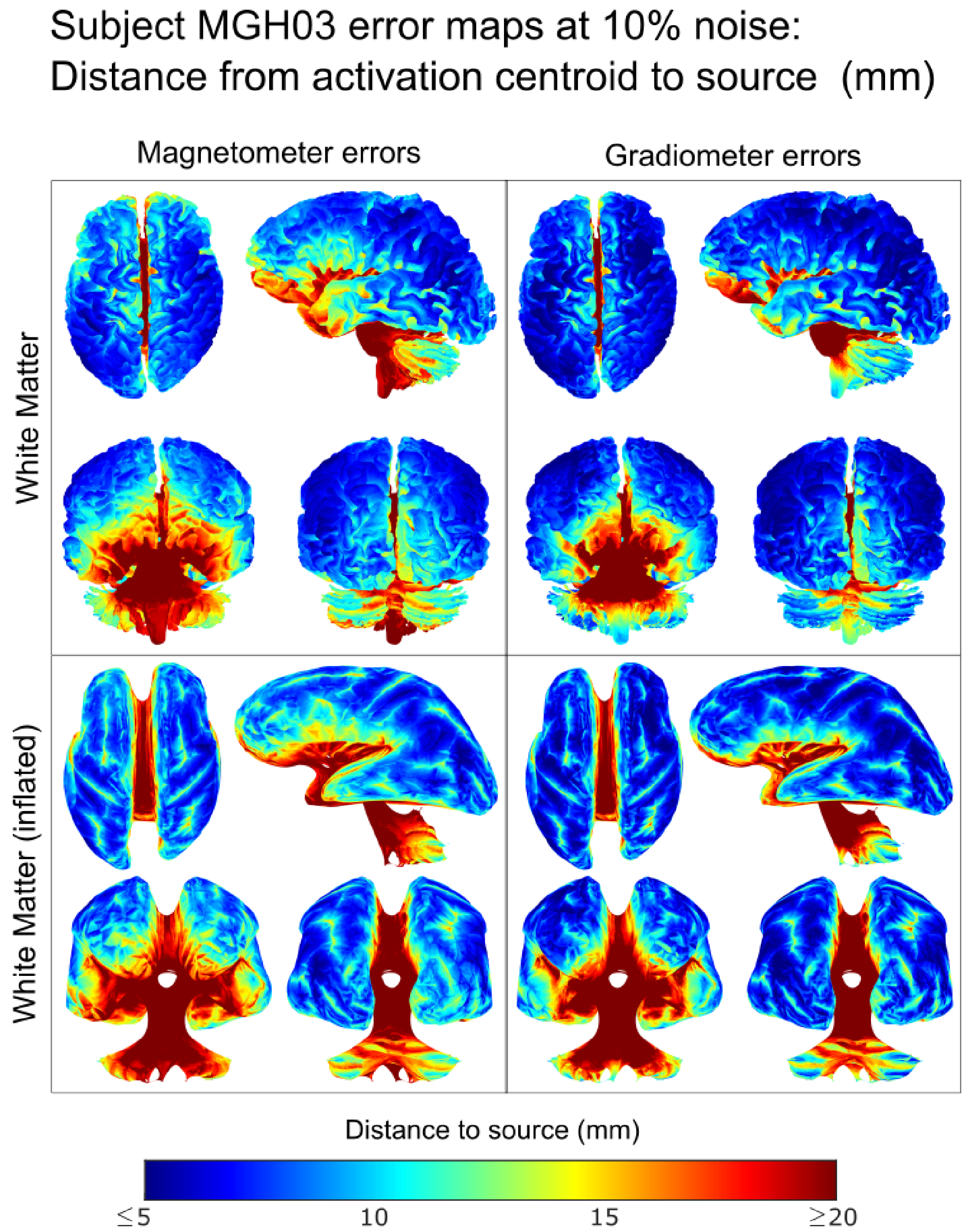

Figure 13: Error maps at 10% noise level (mm) for subject MGH03 on the white matter and inflated white matter surfaces.

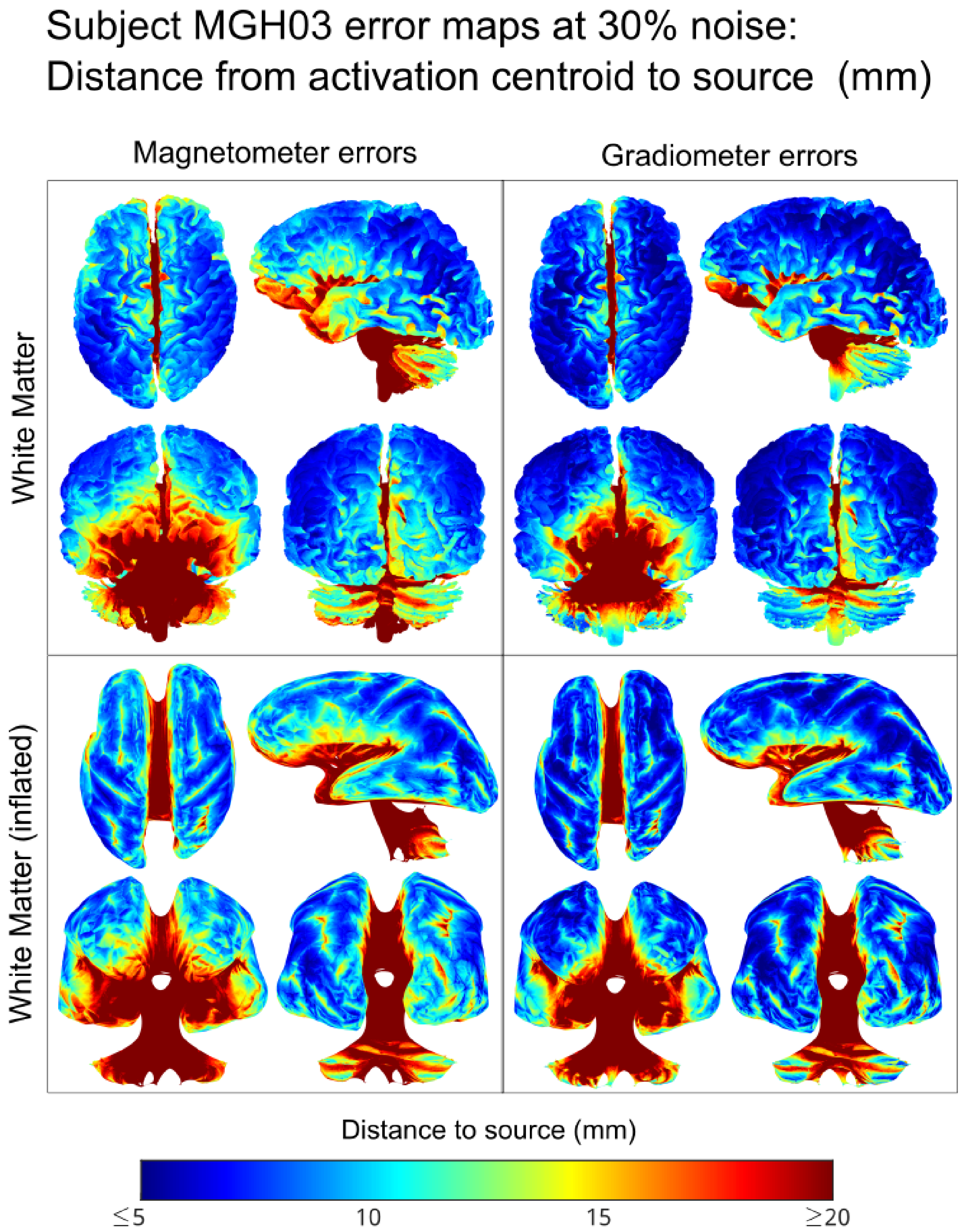

Figure 14: Error maps at 30% noise level (mm) for subject MGH03 on the white matter and inflated white matter surfaces.

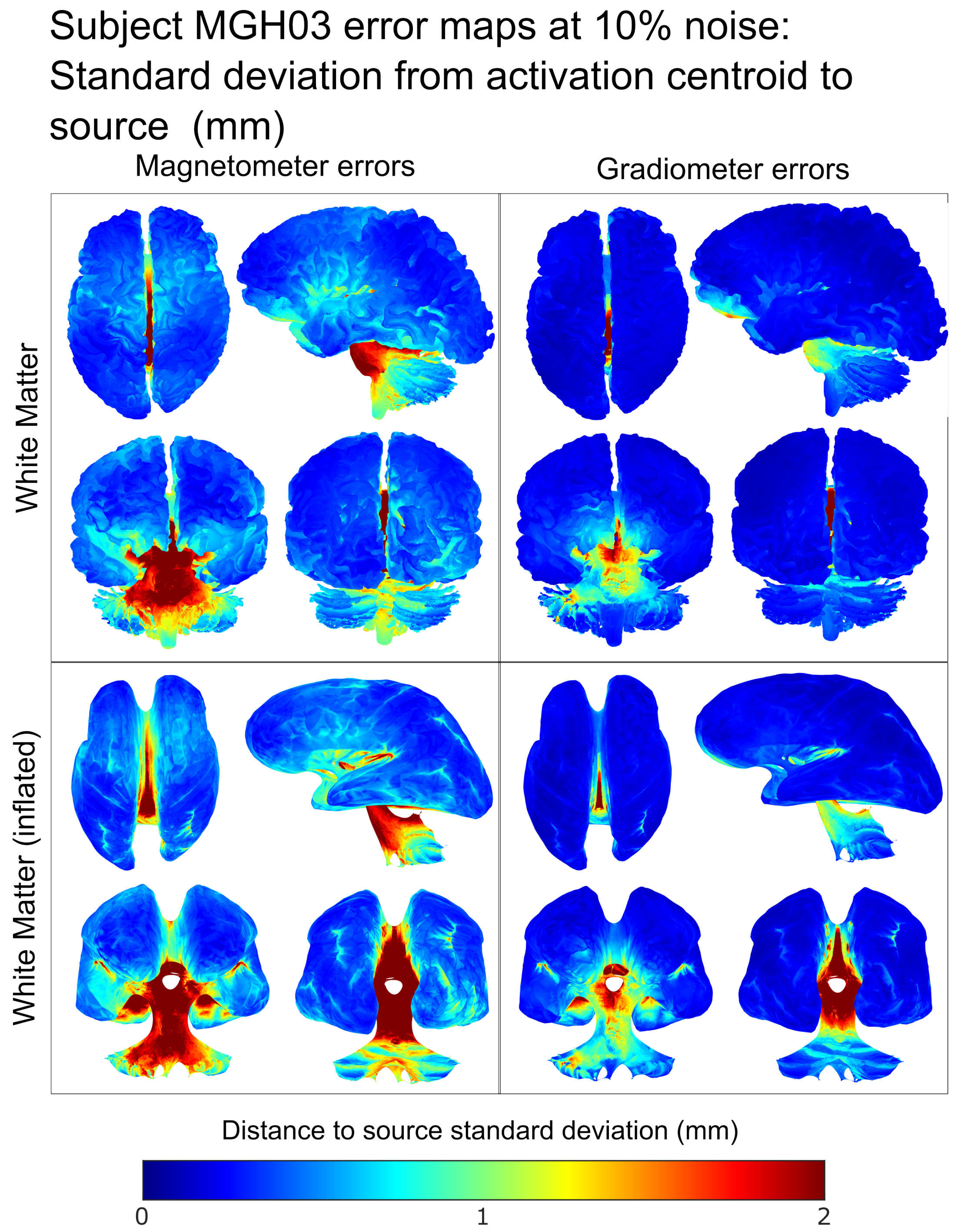

Figure 15: Error maps (standard deviation) at 10% noise level (mm) for subject MGH03 on the white matter and inflated white matter surfaces.

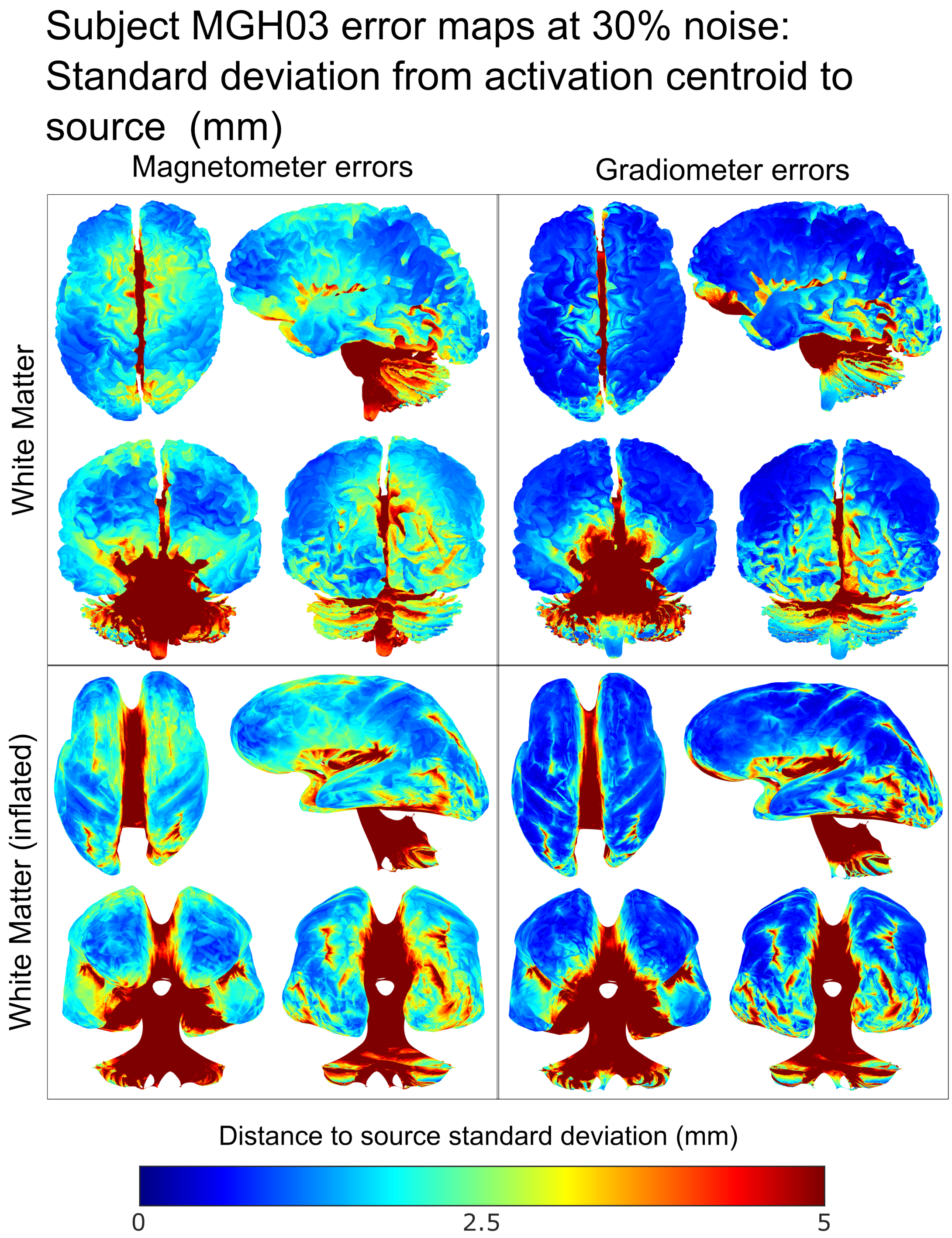

Figure 16: Error maps (standard deviation) at 30% noise level (mm) for subject MGH03 on the white matter and inflated white matter surfaces.

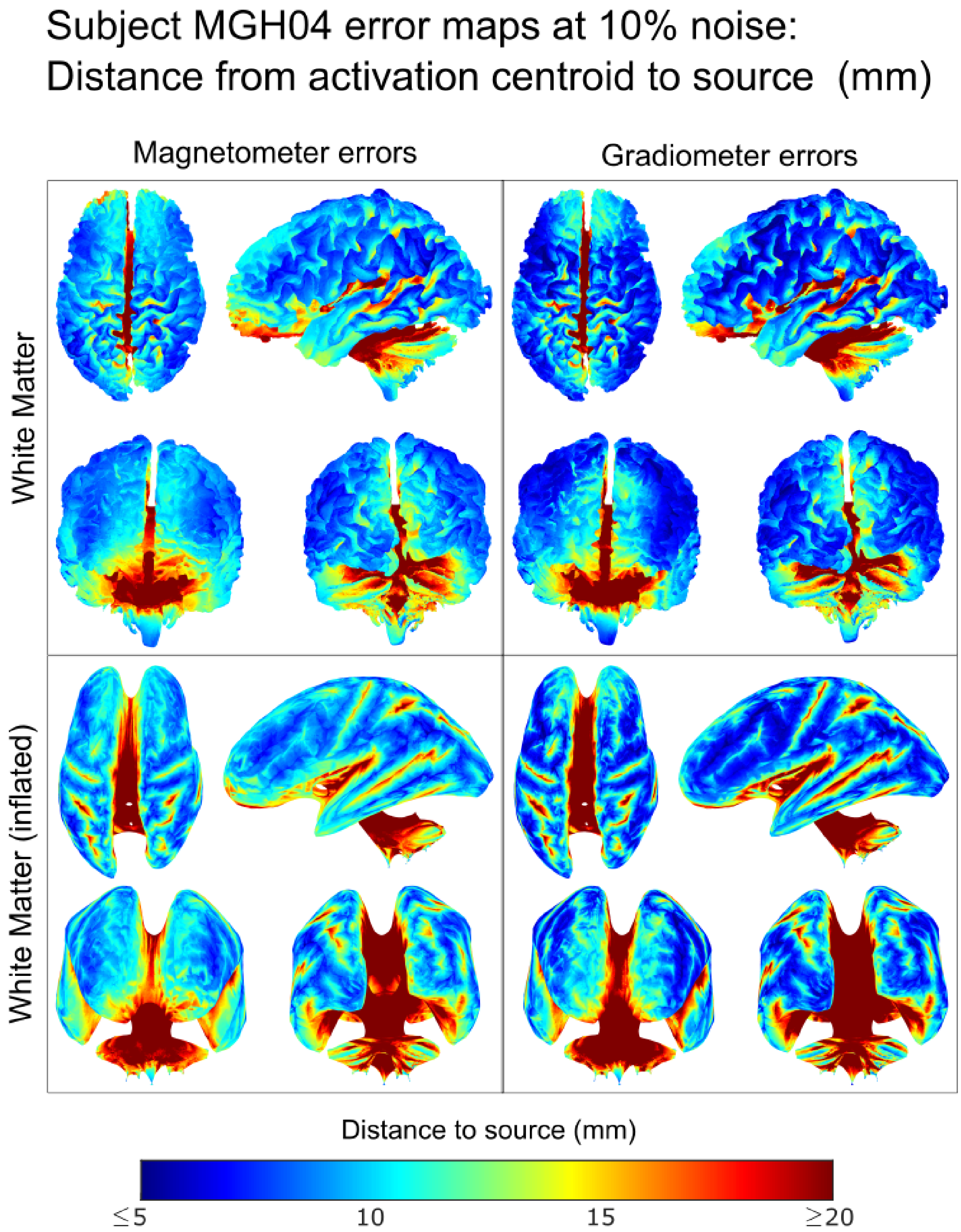

Figure 17: Error maps at 10% noise level (mm) for subject MGH04 on the white matter and inflated white matter surfaces.

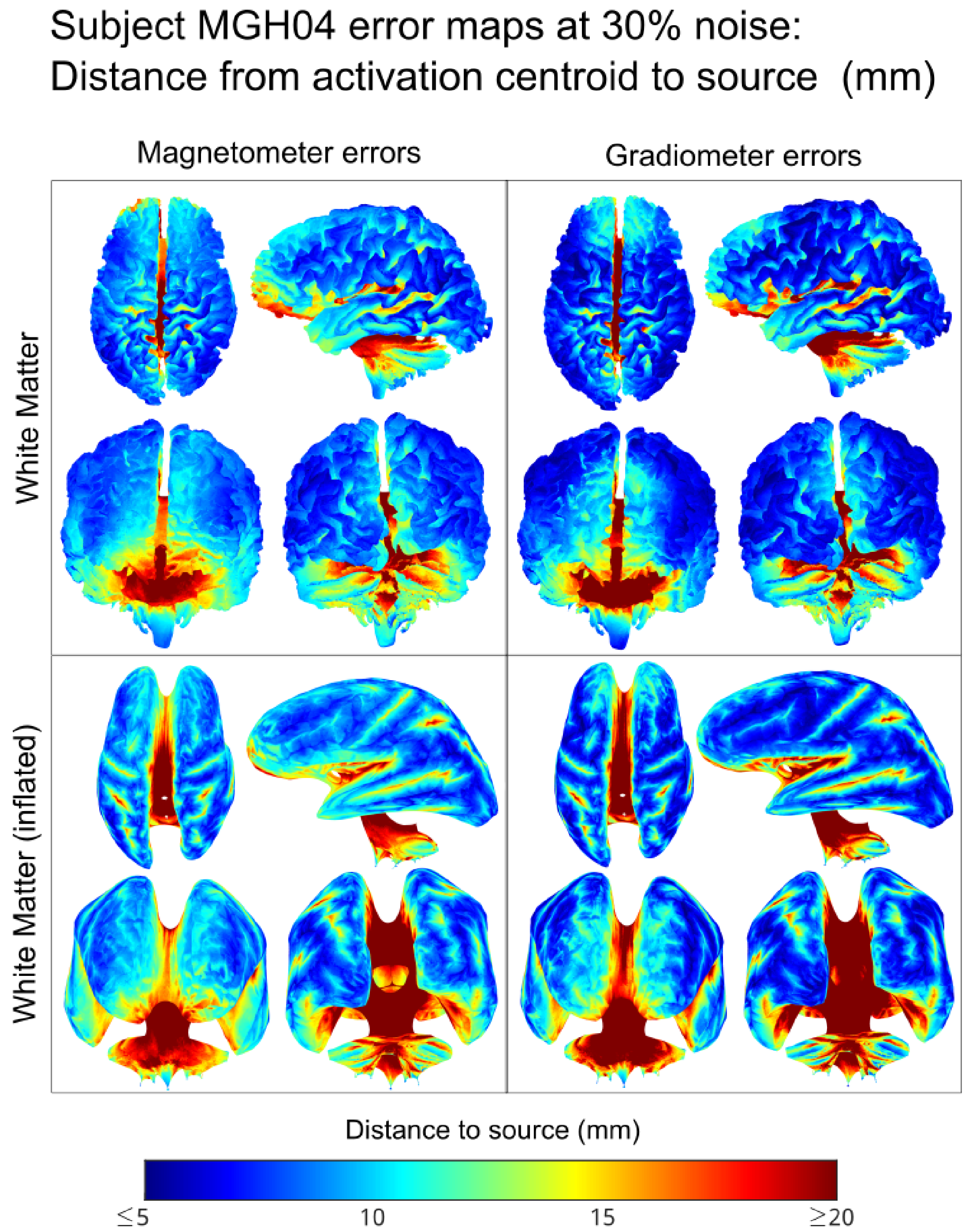

Figure 18: Error maps at 30% noise level (mm) for subject MGH04 on the white matter and inflated white matter surfaces.

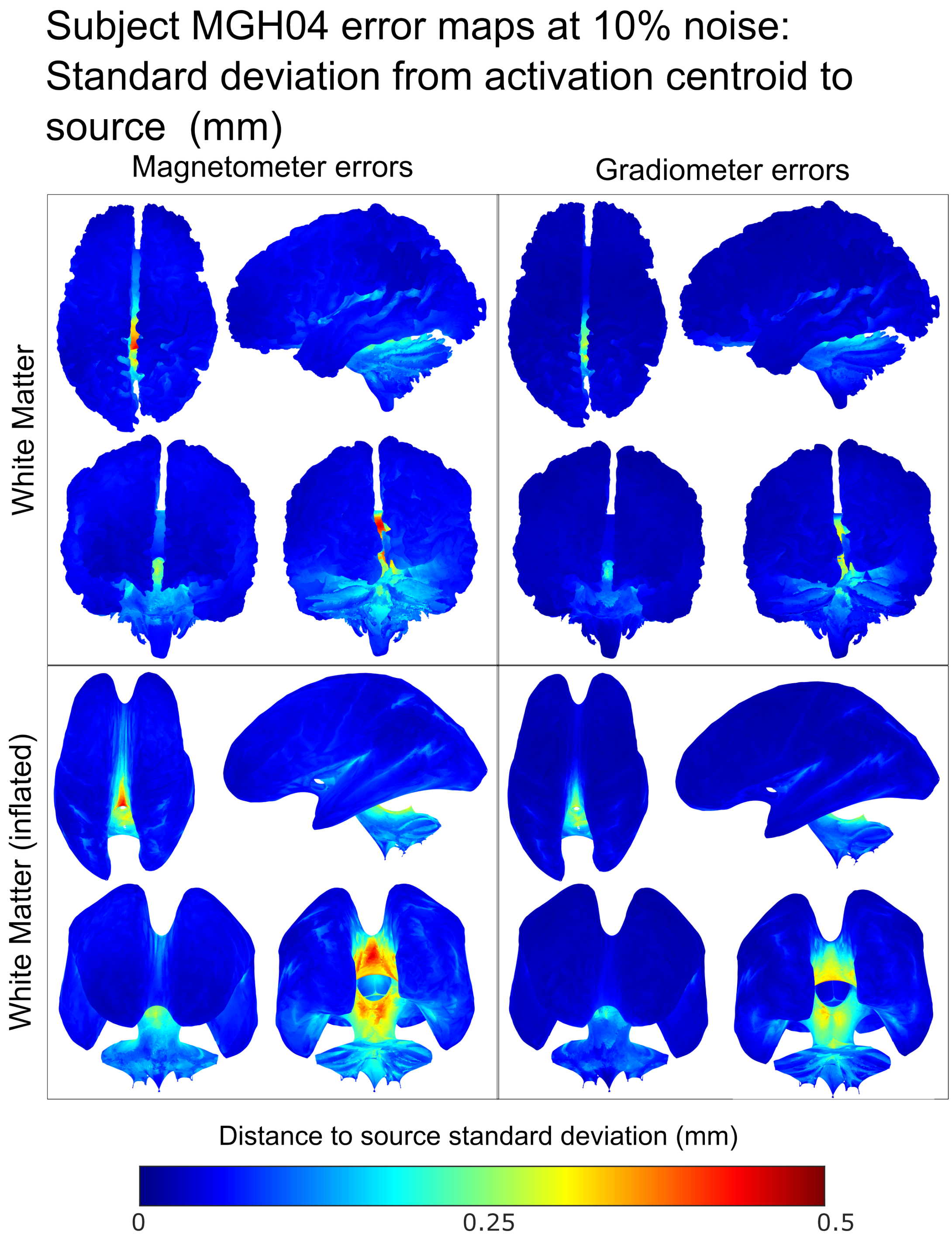

Figure 19: Error maps (standard deviation) at 10% noise level (mm) for subject MGH04 on the white matter and inflated white matter surfaces.

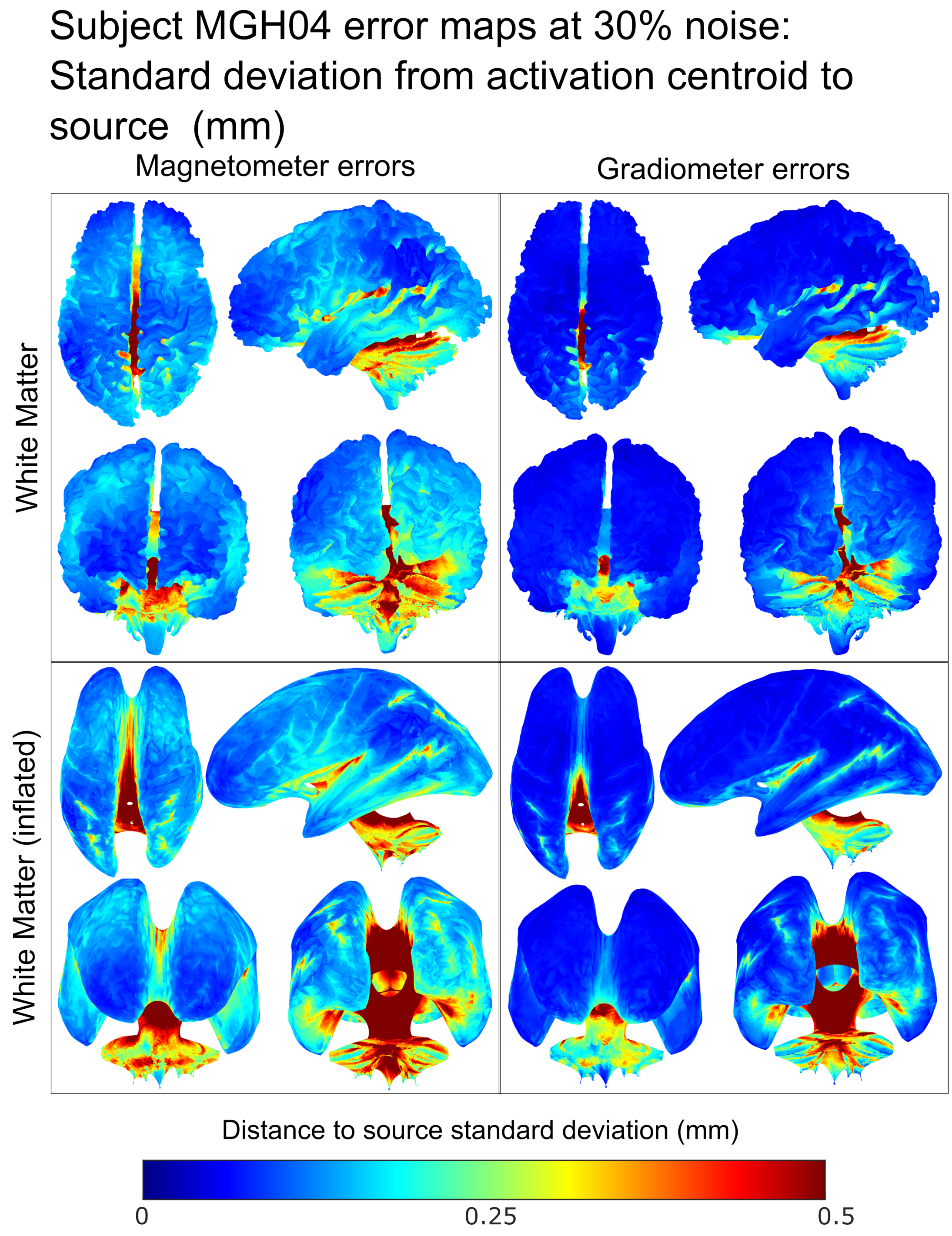

Figure 20: Error maps (standard deviation) at 30% noise level (mm) for subject MGH04 on the white matter and inflated white matter surfaces.

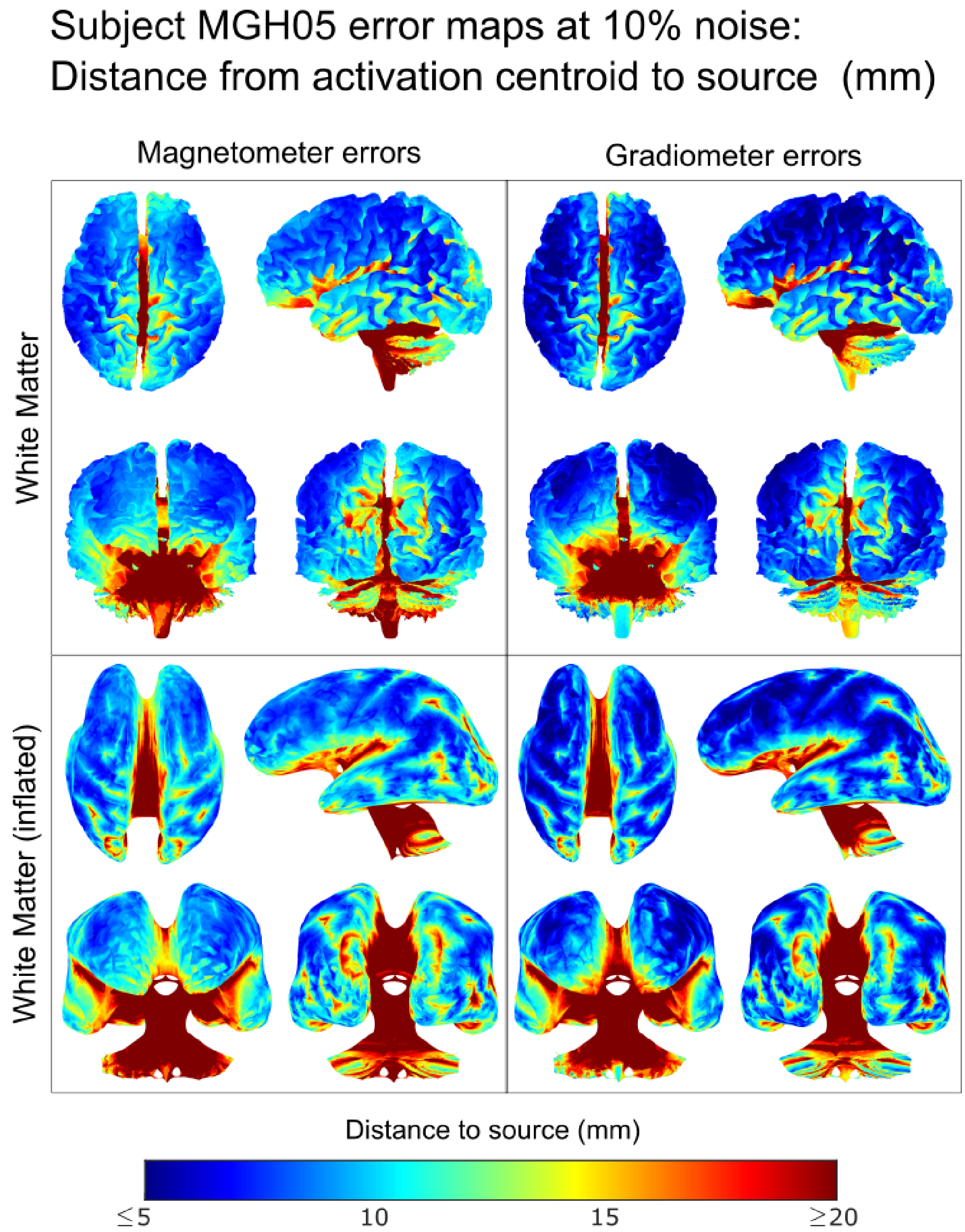

Figure 21: Error maps at 10% noise level (mm) for subject MGH05 on the white matter and inflated white matter surfaces.

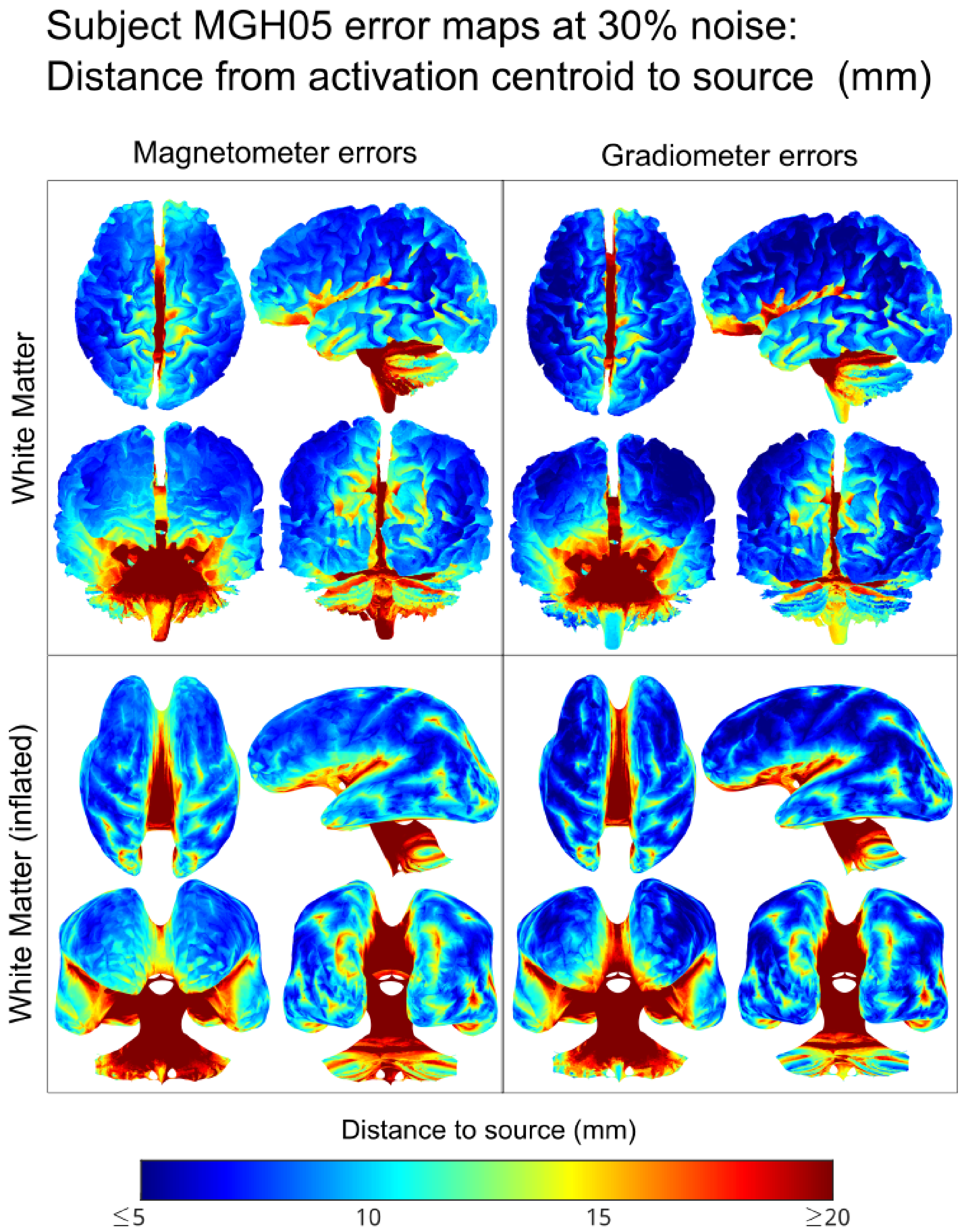

Figure 22: Error maps at 30% noise level (mm) for subject MGH05 on the white matter and inflated white matter surfaces.

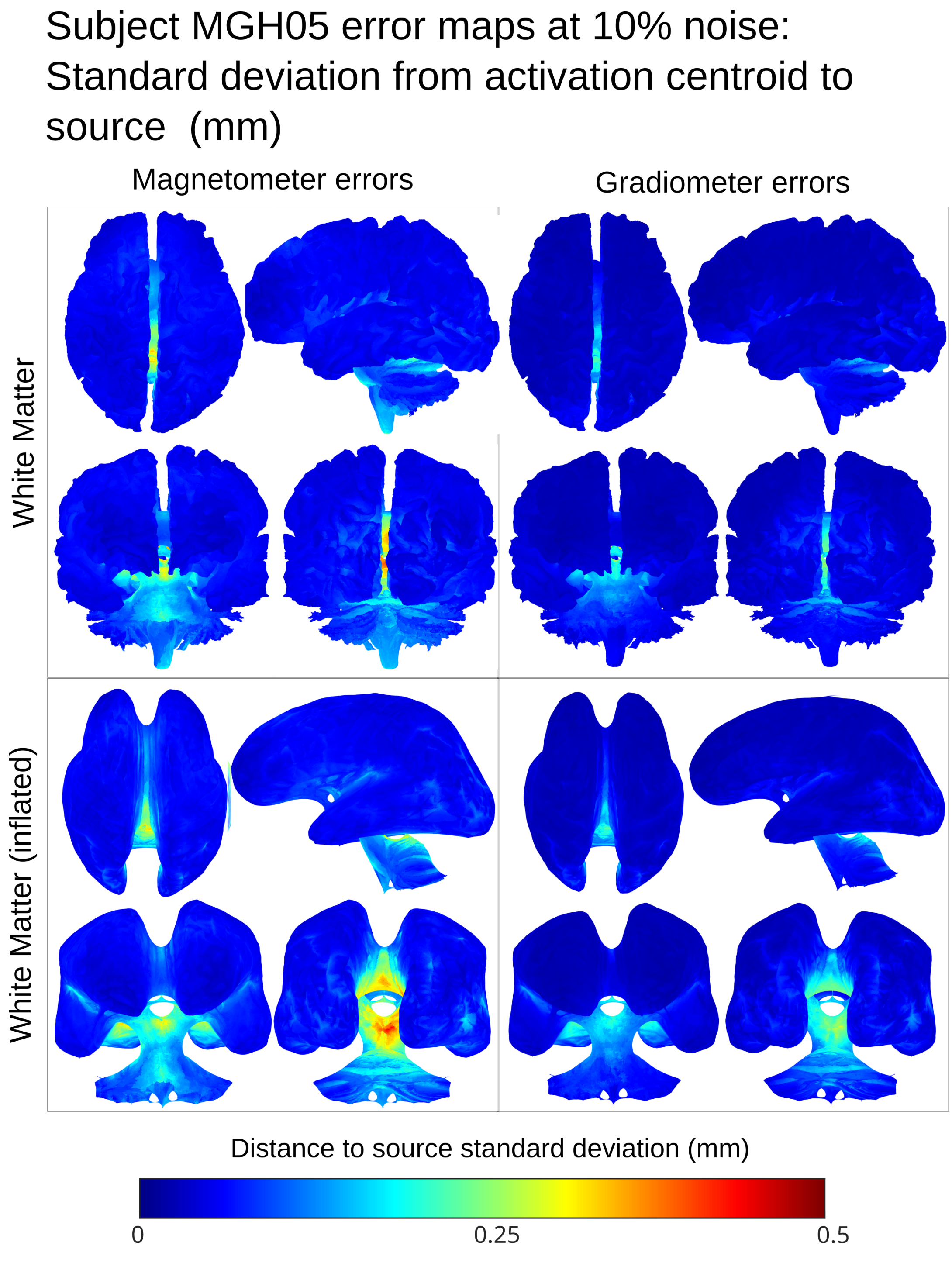

Figure 23: Error maps (standard deviation) at 10% noise level (mm) for subject MGH05 on the white matter and inflated white matter surfaces.

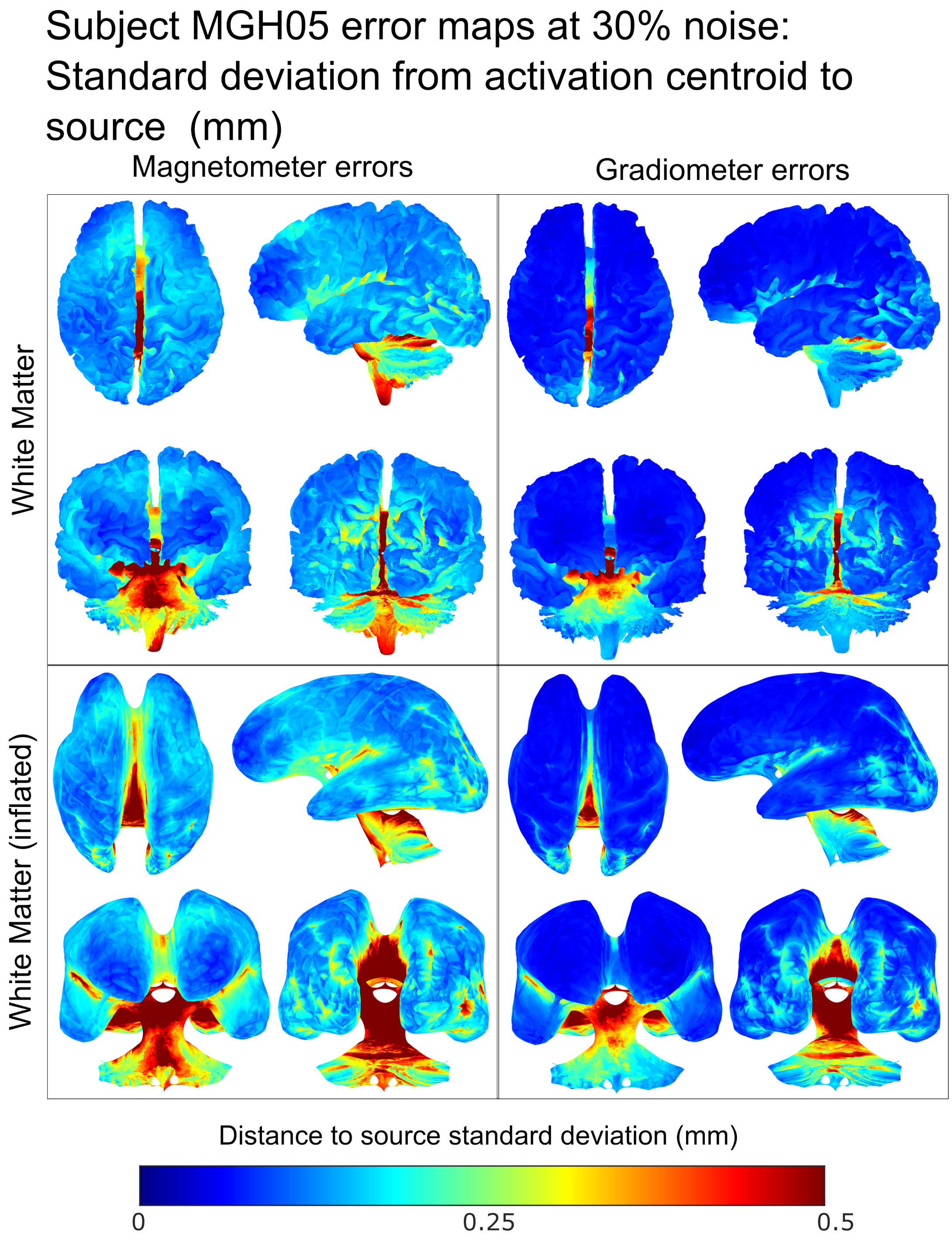

Figure 24: Error maps (standard deviation) at 30% noise level (mm) for subject MGH05 on the white matter and inflated white matter surfaces.
